# Intrapancreatic fat, pancreatitis, and pancreatic cancer

**DOI:** 10.1007/s00018-023-04855-z

**Published:** 2023-07-15

**Authors:** Anna C. Lilly, Igor Astsaturov, Erica A. Golemis

**Affiliations:** 1 Program in Cancer Signaling and Microenvironment, Fox Chase Cancer Center, 333 Cottman Ave., Philadelphia, PA 19111 USA; 2 Molecular & Cell Biology & Genetics (MCBG) Program, Drexel University College of Medicine, Philadelphia, PA 19102 USA; 3 The Marvin & Concetta Greenberg Pancreatic Cancer Institute, Fox Chase Cancer Center, Philadelphia, PA 19111 USA; 4 Department of Cancer and Cellular Biology, Lewis Katz School of Medicine, Philadelphia, PA 19140 USA

**Keywords:** Ductal cells, Cilia, Pancreatic steatosis, PDAC, Lipids

## Abstract

Pancreatic cancer is typically detected at an advanced stage, and is refractory to most forms of treatment, contributing to poor survival outcomes. The incidence of pancreatic cancer is gradually increasing, linked to an aging population and increasing rates of obesity and pancreatitis, which are risk factors for this cancer. Sources of risk include adipokine signaling from fat cells throughout the body, elevated levels of intrapancreatic intrapancreatic adipocytes (IPAs), inflammatory signals arising from pancreas-infiltrating immune cells and a fibrotic environment induced by recurring cycles of pancreatic obstruction and acinar cell lysis. Once cancers become established, reorganization of pancreatic tissue typically excludes IPAs from the tumor microenvironment, which instead consists of cancer cells embedded in a specialized microenvironment derived from cancer-associated fibroblasts (CAFs). While cancer cell interactions with CAFs and immune cells have been the topic of much investigation, mechanistic studies of the source and function of IPAs in the pre-cancerous niche are much less developed. Intriguingly, an extensive review of studies addressing the accumulation and activity of IPAs in the pancreas reveals that unexpectedly diverse group of factors cause replacement of acinar tissue with IPAs, particularly in the mouse models that are essential tools for research into pancreatic cancer. Genes implicated in regulation of IPA accumulation include KRAS, MYC, TGF-β, periostin, HNF1, and regulators of ductal ciliation and ER stress, among others. These findings emphasize the importance of studying pancreas-damaging factors in the pre-cancerous environment, and have significant implications for the interpretation of data from mouse models for pancreatic cancer.

## Introduction

Rates and degree of obesity have been rising across the globe for several decades; as of 2016, 39% of adults were classified as overweight and 13% as obese [1, 2]. In the United States, a 2017–2018 survey estimated that 31.1% of adults are overweight, and over 42% of adults are obese [3]. Obesity causes complex changes in multiple organ systems, but is typically characterized by adipocyte expansion, fat deposits, immune cell recruitment, and low-grade chronic inflammation [2, 4, 5]. These changes are associated with increased risk for and exacerbation of diseases affecting multiple organ systems.

Incidence of diseases of the pancreas, including acute and chronic pancreatitis [6, 7] and pancreatic cancer [8], are rising globally, with the highest rates of these diseases observed in North America and Europe. These rising rates have been linked to a number of modifiable factors, including increased preference of individuals for high calorie and high fat diets that promote obesity; the role of obesity in inducing and exacerbating pancreatitis [9, 10] and pancreatic cancer [11–15] has been appreciated for over a decade. It has been suggested that common pathological changes underlie the development of both chronic pancreatitis and pancreatic cancers, given that chronic pancreatitis is well-established as a risk factor for subsequent development of pancreatic cancer [16]. With over 73% of U.S adults and 52% of adults worldwide categorized as overweight or obese, and with pancreatic cancer one of the most lethal forms of cancer [8], understanding the mechanisms by which obesity affects the onset and progression of pancreatic diseases has become a priority.

Obesity causes both endocrine and paracrine effects on cell function in the pancreas. Endocrine effects have been extensively reviewed, arise from both visceral adipose tissue and intra-organ fat deposition, and include elevated production of sex hormones, and increased production of insulin [17, 18]. Paracrine effects, which include adipocyte secretion of pro-inflammatory cytokines and adipokines, are more commonly associated with local ectopic intra-organ accumulation of fat cells and intracellular accumulation of lipid droplets [19]. There is growing evidence that intra-organ adipocyte accumulation is particularly pernicious, creating an unhealthy metabolic environment that is specifically disease-promoting, causing focus on the factors supporting generation and function of localized deposits of fat tissue [20]. Beyond obesity, aging also is associated with an increased risk of diseases of the pancreas [8], and many aging-related changes in the pancreas overlap with those associated with obesity, including increased inflammation and replacement of the normal tissue mass with fat cells [21, 22]. Together with obesity, the rapid aging of global populations creates a strong risk environment for pancreatic disease, emphasizing the need to characterize and develop approaches to reverse sources of risk.

For some tissues, e.g., cardiac and skeletal muscle [20, 23], the mechanisms of generation of intra-organ adiposity are becoming well-defined. In contrast, the mechanistic basis by which fat accumulates and then interacts with the exocrine and endocrine cells of the pancreas to increase their propensity to undergo pathological changes is very poorly defined, despite numerous clinical studies have linked accumulation of pancreatic fat with pathological features of pancreatitis and pancreatic cancer. As such lack of clarity poses challenges to strategies to target and reverse the rising rates of pancreatic disease, our goal in this review has been to analyze the literature relevant to the role and etiology of intra-pancreatic adipocytes (IPAs) in pancreatic dysfunction. Intriguingly, although there is no integrated model for the origins of IPAs, a number of genetic studies have suggested mechanisms and precursor cells contributing to IPA accumulation. We discuss the process of pancreatic fat accumulation in the context of evidence indicating reprogramming of signaling pathways typically associated with pancreatic development as a potentially targetable contributor to the process of obesity-promoted pancreatic pathogenesis.

### Pancreatitis

**Fig. 1:**
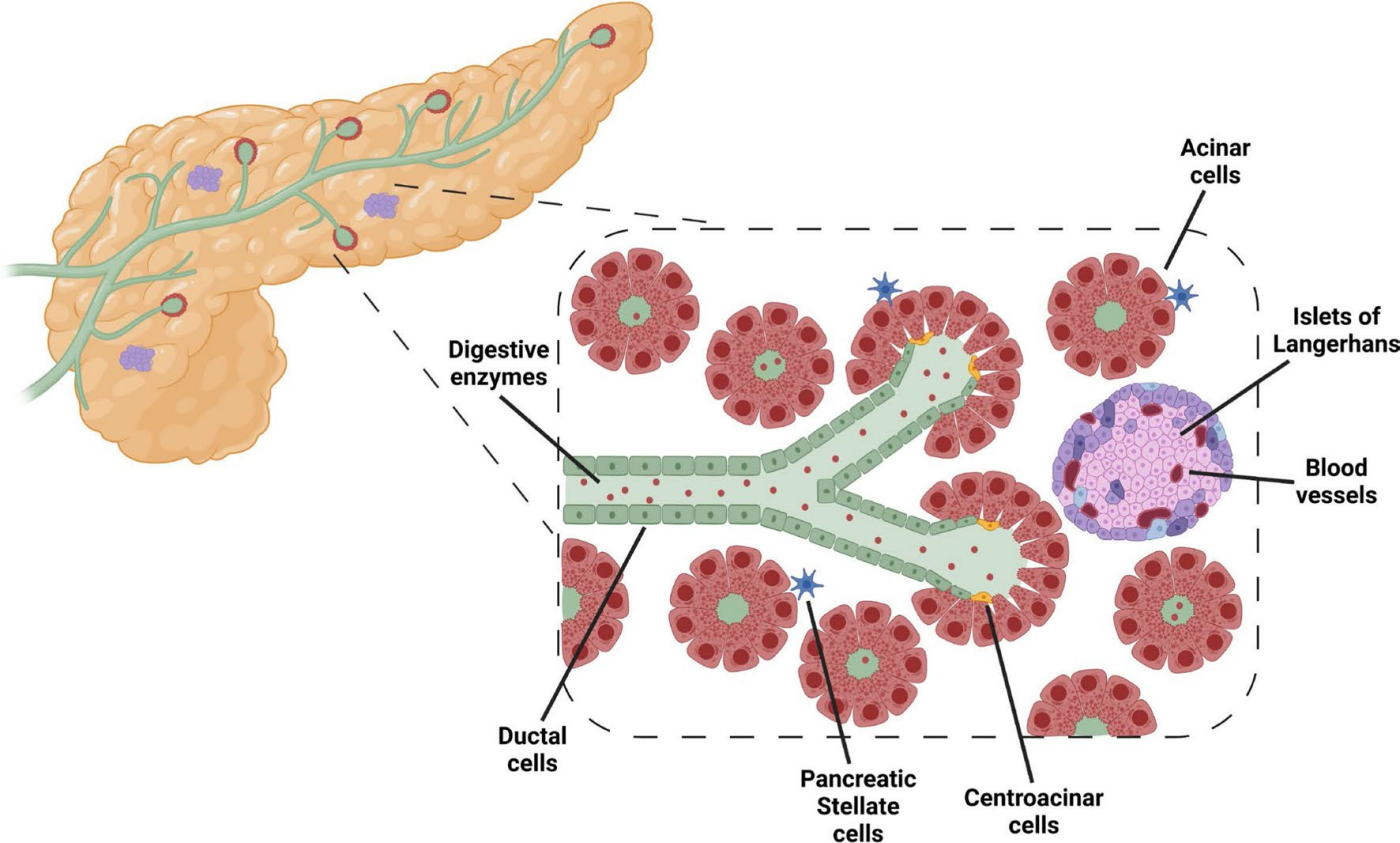
Normal pancreatic structure. A normal pancreas consists of two main compartments, the endocrine and exocrine. The endocrine compartment consists of hormone-producing cells found within Islets of Langerhans, which produce insulin and glucagon to regulate blood glucose levels throughout the body. The exocrine compartment is composed of acinar cells, ductal cells, and centroacinar cells, and represents the bulk of the pancreas. Acinar cells, organized in structures called acini, produce digestive enzymes that are transported by ducts to the duodenum to aid in digestion. Centroacinar cells are specialized cells that extend from the duct into pancreatic acini, and play a role in pancreatic regeneration. Pancreatic stellate cells (PSCs) are myofibroblast-like cells found in both pancreatic compartments that contribute to tissue modeling, secrete extracellular matrix, and can be a source of cytokines. Created with BioRender.com

The adult pancreas is divided into lobules, separate by thin layers of connective tissue. The microanatomy of the pancreas reflects its dual exocrine and endocrine role (Fig. 1). The exocrine function involves the production of digestive enzymes such as amylase, lipase, and trypsin by acinar cells, which represent ~ 85% of the pancreatic mass, and are the major constituents of the lobules. The acinar cells are organized in clusters around small intercalated ducts, and secrete the enzymes (stored in inactive form as zymogen granules) into these ducts; ducts merge into larger intralobular, then interlobular ducts, and eventually drain into the duodenum. The endocrine function is mediated by cells organized in small pancreatic islets (the islets of Langerhans) which consist of several types of endocrine cells (most important are α, which produce glucagon; β, which produce insulin; and δ, which produce somatostatin); these islets are dispersed throughout the pancreas, and drain directly into arterioles. Other resident cell populations include centroacinar cells, located at the tips of the ducts, characterized by features both of acinar and embryonic stem cells; and pancreatic stellate cells (PSCs), fibroblast-like cells that help maintain the extracellular matrix.

**Fig. 2:**
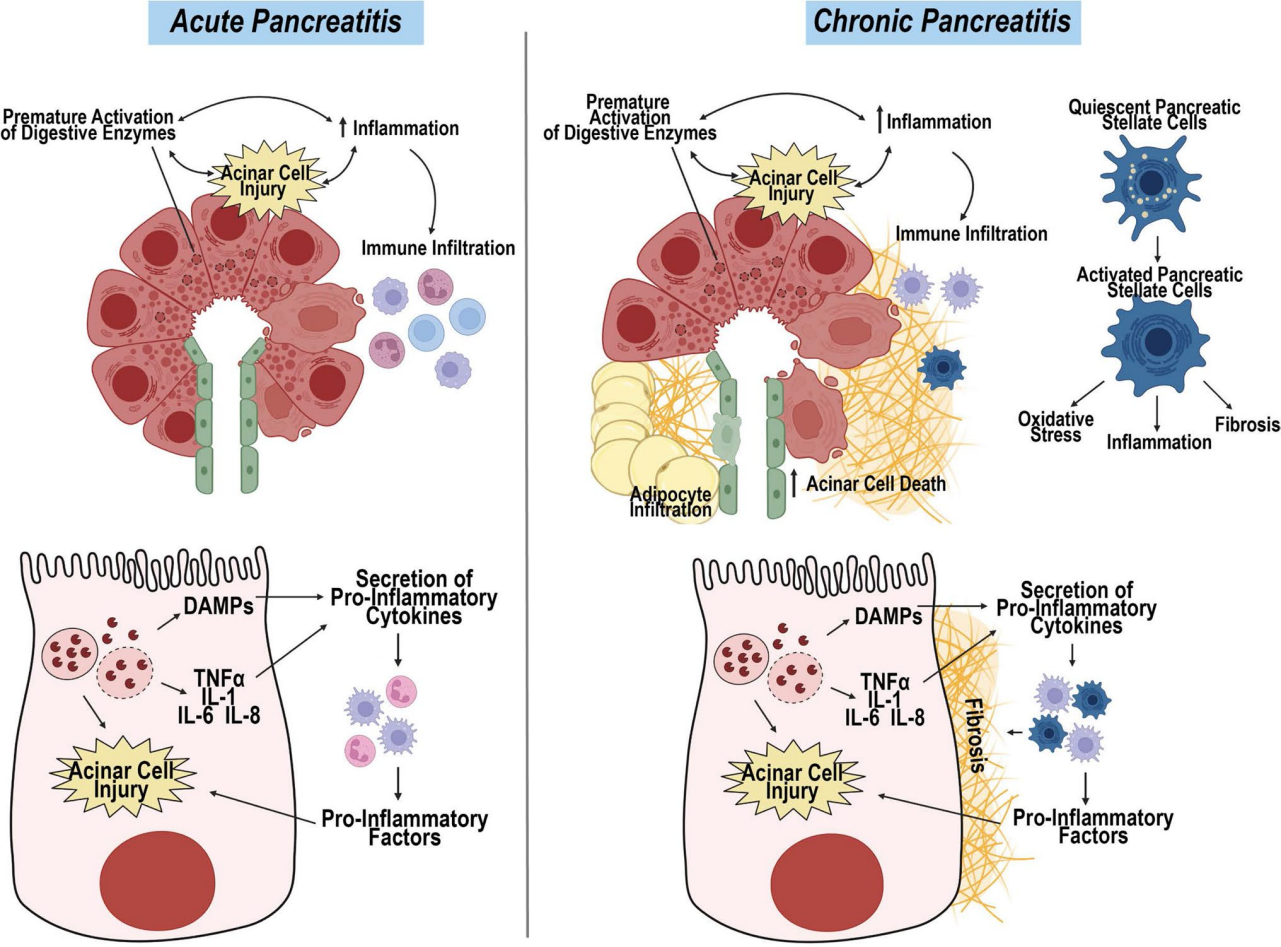
Pathophysiology of acute and chronic pancreatitis. **A** Acute pancreatitis is acinar injury and pancreatic inflammation caused by mechanical obstruction of ducts, or systemic factors that trigger premature activation of digestive enzymes. In acinar cells, damage induced by these stressors increases expression of damage-associated molecular patterns (DAMPs), TNFα, IL-6, and IL-8, which are secreted into the local microenvironment and promote infiltration of leukocytes such as lymphocytes, granulocytes and macrophages. Infiltrating immune cells secrete their own pro-inflammatory factors, increasing acinar cell injury and acinar cell death. **B** Chronic pancreatitis describes prolonged inflammation caused by a variety of factors, including recurrent severe acute pancreatitis. Similar to acute pancreatitis, acinar cell injury drives expression of pro-inflammatory cytokines that promotes immune infiltration. Unlike acute pancreatitis, macrophages make up the majority of the immune landscape in chronic pancreatitis cases. In addition to immune infiltration, chronic inflammation and cellular stress causes pancreatic stellate cells to activate, causing increased oxidative stress, inflammation and fibrosis that cumulative increase acinar cell death and, in some cases, promote adipocyte infiltration. Created with BioRender.com

Acute pancreatitis (Fig. 2A) describes an event typically fulminant in onset, and often clinically dramatic. The associated pancreatic necrosis and inflammation [24, 25] are triggered by mechanical obstruction of pancreatic or bile ducts blocking efflux of digestive enzymes, or systemic factors responsible for acinar cell damage such as obesity, alcohol use, hypercalcemia, increased blood triglycerides, overproduction of acinar-stimulatory hormones (e.g., cholecystokinin), and certain medications with direct potential for pancreatic toxicity. These ductal and acinar cell defects cause premature release and intracellular activation of potent proteases and lipases which causes a cascade of extensive intrapancreatic tissue damage, and in severe cases can rapidly progress to systemic toxicity. Acute pancreatitis is often accompanied by extensive pancreatic inflammation marked by secretion of pro-inflammatory factors (TNFα, interleukins IL-1, -6, and -8, and damage associated molecular patterns (DAMPs)) from injured pancreatic epithelium and secondary tissues, and by infiltration of macrophages and neutrophils, which release additional pro-inflammatory factors into the circulatory system that can further contribute to localized tissue damage, and promote multi-organ failure. Notably, accumulation of IPAs can promote this inflammatory process. These adipocytes provide a rich source of triglycerides, that are processed by lipases released from damaged acinar cells to unsaturated free fatty acids (including linoleic, linolenic, and oleic acids) that inhibit mitochondrial function and enhance cytokine release, thus exacerbating acinar cell injury and contributing to organ failure [9, 26–29].

If the primary triggers of acute pancreatitis (e.g., use of medications or alcohol) are not identified or mitigated, recurrent episodes of acute pancreatitis can lead to chronicity of the disease. Estimates of how many individuals who have had acute pancreatitis who subsequently develop chronic pancreatitis vary considerably, ranging from 3 to 35% [7, 25]. Although acute pancreatitis is a predisposing condition for chronic pancreatitis (Fig. 2B), not all individuals developing chronic pancreatitis [7, 30] have well-documented cases of acute pancreatitis [30]. Although alcohol is a primary risk factor, other well-appreciated risk factors include genetic predisposition and tobacco smoking. Inherited gene variants that impose risk (in genes including *PRSS1, SPINK, CTRC, CFTR*, and *CPA1*) typically result in premature activation of digestive enzymes in acinar cells, and have been linked to chronic pancreatitis. Early stages of chronic pancreatitis include damage to acinar cells and development of localized inflammation. Repeated cycles of damage, and activation of pancreatic fibroblasts (*aka,* stellate cells, or PSCs) to a myofibroblast-like phenotype (promoted by smoking) distorts the morphology of the pancreas, marked by accumulation of fibrosis and IPAs in the setting of ongoing inflammation. Over time, these changes give rise to exocrine and endocrine insufficiency due to loss of acinar mass and reduction in number and function of islets, resulting in incomplete digestion of foods in the intestinal tract, steatorrhea, bacterial overgrowth, malabsorption syndrome and type 2 diabetes.

### Pancreatic cancer

**Fig. 3:**
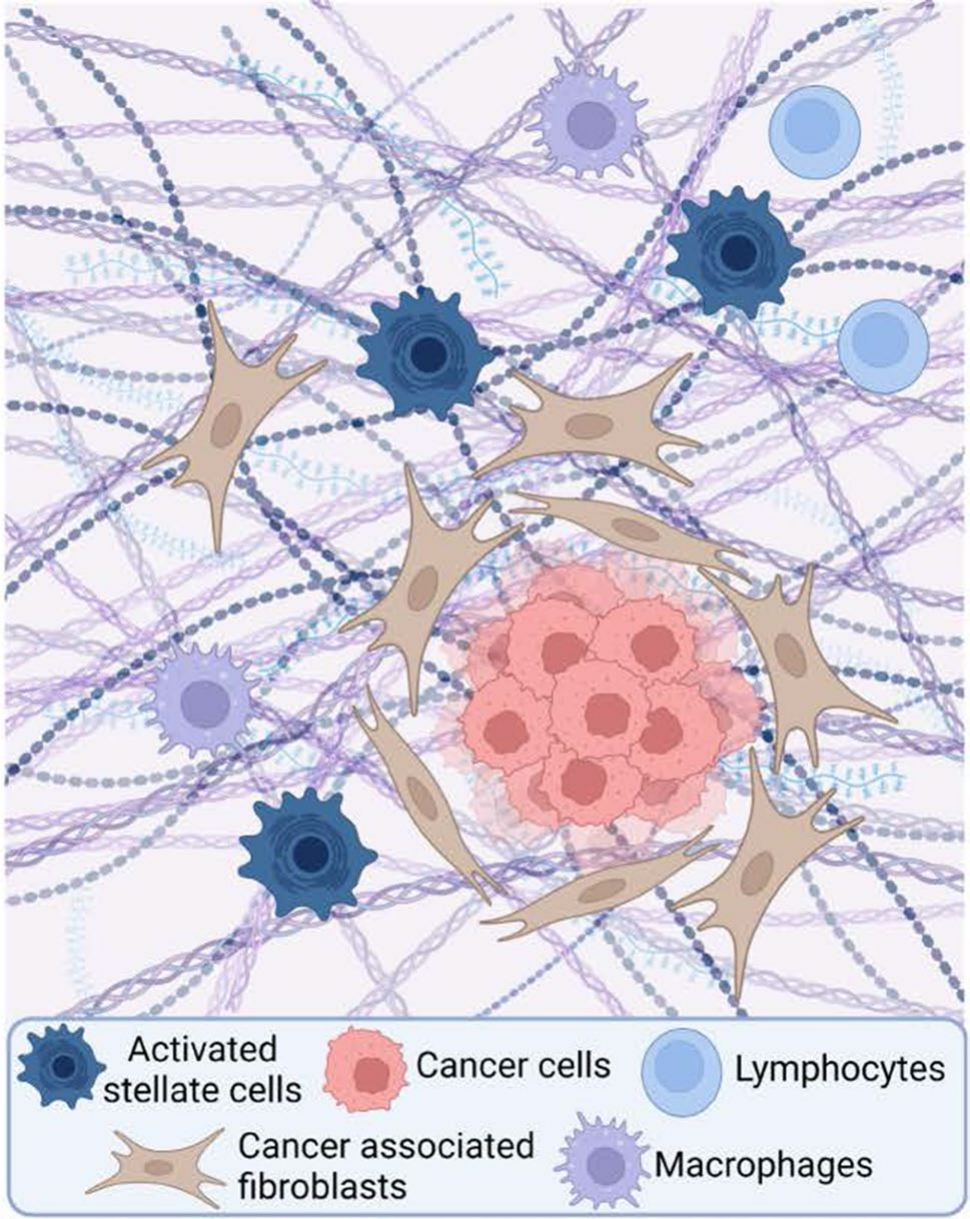
Pancreatic cancer. Pancreatic cancer area consists of relatively small numbers of cancer cells within the tumor mass. The majority of the tumor is stroma that consists of extracellular matrix, cancer associated fibroblasts (CAFs), activated pancreatic stellate cells, and tumor promoting immune cells (macrophages and lymphocytes). Created with BioRender.com

The most common cancer of the pancreas is pancreatic ductal adenocarcinoma (PDAC) (Fig. 3), with neuroendocrine tumors and acinar carcinomas occurring at much lower frequencies. Modifiable risk factors for PDAC include chronic pancreatitis (both alcohol-associated and independent), which elevates risk of PDAC by tenfold; obesity, which includes accumulation of IPAs, visceral fat, tobacco smoking, and diabetes [31]. Inherited risk factors include gene variants damaging in PRSS1 and CFTR (both also associated with chronic pancreatitis), and tumor suppressors involved in DNA repair processes (including *BRCA1, BRCA2, PALB2, MLH1, CDKN2A*, and *ATM*). Individuals with Peutz-Jeghers syndrome, associated with mutations inactivating the *STK11* tumor suppressor gene, are at high risk for pancreatic cancer [32–34]. *STK11* encodes LKB1, which signals through AMPK to negatively regulate lipid, cholesterol, and glucose metabolism [35]; metabolic changes associated with obesity downregulate the LKB1-AMPK signaling axis, contributing to cancer risk.

Over 90% of PDACs contain somatic activating mutations in KRAS, with mutations in tumor suppressor genes *TP53, CDKN2A*, and *SMAD4* observed in 50–80% of PDAC cases. As with many tumors, emergence of an established carcinoma is accompanied by the epigenetic and metabolic promoting to enhance tumor growth and survival, in part by promoting immunosuppression. An unusual feature of PDAC is an intense desmoplastic reaction, in which PSCs become activated to cancer-associated fibroblasts (CAFs); altered and elevated secretion of extracellular matrix (ECM) components by the CAFs results in formation of a dense fibrotic mass that surrounds growing PDACs [36]. While this matrix can be tumor-promoting (for instance, by limiting access of tumor-suppressive lymphocytes to the cancer cells), it contributes to a nutrient-poor, hypoxic environment; as a result, PDACs are highly dependent on autophagy [37] and macropinocytosis [38] for uptake of nutrients from the tumor microenvironment. Typically, at the stage of diagnosed PDAC, few IPAs are detected within the tumor mass.

The clinical manifestation of human PDAC is preceded by precursor lesions which may be present for months to years until evolving to invasive malignancy [39, 40]; during this period, the precursor lesions often exist in a microenvironment characterized by IPAs, fibrosis, and inflammation. Many studies propose, and considerable data supports, the idea that acinar-to-ductal metaplasia (ADM)—the epigenetic reprogramming of acinar cells to partially de-differentiate, then acquire ductal features such as the expression of CK19 and SOX9 [41]—is an important and irreversible early step for the emergence of microscopic lesions known as pancreatic intraepithelial neoplasms (PanINs) [15, 42]. This trajectory is thought to be the dominant origin for PDAC formation. However, in a significant proportion of patients, PDAC can emerge from pre-existing larger lesions arising from expansion of ductal epithelium- mucinous cystic neoplasm (MCN) and intraductal papillary mucinous neoplasm (IPMN).

The specific cell of origin of PanINs has been a topic of considerable interest [43]. Although PDACs are thus named because they contain features of pancreatic ductal cells, studies with mouse models have demonstrated that specific expression of activated KRAS in acinar and centroacinar cells in embryos can lead to formation of PanINs and PDAC (e.g. [44]). Complicating the picture, other studies using mouse models with cell lineage-specific promoters have shown that ductal cells and endocrine cells can also form PanINs, in the context of specific additional mutations or the inflammatory environment associated with chronic pancreatitis [45–47]. One study using inducible promoters to activate KRAS specifically in GFP-tagged adult acinar and ductal cells found oncogenic KRAS induced PanIN and PDAC formation in acinar but not ductal cells within 2 months, while simultaneous activation of KRAS with loss of TP53 in ductal cells was required to cause formation of PDAC, albeit without associated PanINs [46].

These differing results implied that there are multiple independent routes to forming PDACs, involving distinct cells of origin. An elegant set of analyses using mouse models to investigate the interaction of *KRAS* and distinct *TP53* mutations expressed in acinar versus ductal cells has confirmed the specific importance of *KRAS* mutations in promoting PDAC in ductal cells [43]. Careful dissection of tumor phenotypes in this study convincingly demonstrated that those originating from ductal cells have many features resembling the basal cell type, while those originating from acinar cells that subsequently undergo ADM resemble the classical subtype [43]. Notably, the advent of transcriptomic profiling and single cell sequencing studies has helped define the existence of distinct subtypes of pancreatic cancer, with distinct prognoses, including the classical (glandular) and basal (mesenchymal) subtypes of PDAC [48]. Moreover, there is growing appreciation for intratumoral differentiation heterogeneity within these PDAC subtypes, as well as continuous differentiation plasticity within normal acinar [49] and ductal [50] cells containing multiple populations with progenitor features to terminally differentiated states. This considerable plasticity of acinar [15] and other pancreas-resident cells is critical in maintaining normal homeostasis, repairing tissue damage, but also provides diverse substrates for oncogenic transformation.

Pancreatitis can influence the availability of cell populations that are precursors to oncogenic transformation, while additionally providing inflammatory stimuli that enhance tumor growth. For example, an early study using mouse models demonstrated that endogenous expression of activated KRAS in embryonic acinar cells was sufficient to produce PDAC, but similar expression of activated RAS in the acinar cells of adult mice did not, except in the context of induced chronic pancreatitis [51]. The ability of adult acinar cells expressing active KRAS to form PanINs was less clear [51, 52]. Recent single cell RNA sequencing studies have been informative, comparing populations of acinar cells in the healthy pancreas versus in the context of chronic pancreatic injury. These studies have identified multiple sub-groups of acinar cells, which can transdifferentiate into alternative cell lineages following injury; notably, these include mucinous precursors that can give rise to PanINs in the context of a *KRAS* mutation, as well as ductal precursors [49, 53, 54].

Injury-induced inflammation in the microenvironment of the nascent cancer promotes tumorigenesis. This inflammation may be linked to external factors, such as the tissue damage from pancreatitis, or related to specific predisposing mutations in the cancer cell that promote a pro-inflammatory environment by promoting secretion of cytokines, and inducing endoplasmic reticulum (ER) stress [55, 56]. A key question is how obesity and aging interact with these factors to create a favorable climate for progression of a transformed cancer cell to a progressive tumor. A number of recent studies have emphasized how changes in hormonal and endocrine activities are associated with obesity. Most of these emphasize hormonal and endocrine effects [4], including some operating in the microenvironment of nascent tumors [12]. Along with obesity, aging has been independently associated with a significant increase in IPAs and decline in acinar cell mass [57]. Suggestively, studies of pre-malignant pancreatic lesions (PanINs and IPMNs) in aged or obese individuals, or those genetically predisposed to pancreatic cancer, have indicated these lesions occur proximal to areas of parenchymal atrophy and IPAs [58, 59].

### Accumulation of intra-pancreatic adipocytes and lipid droplets in pancreatitis and cancer

**Fig. 4:**
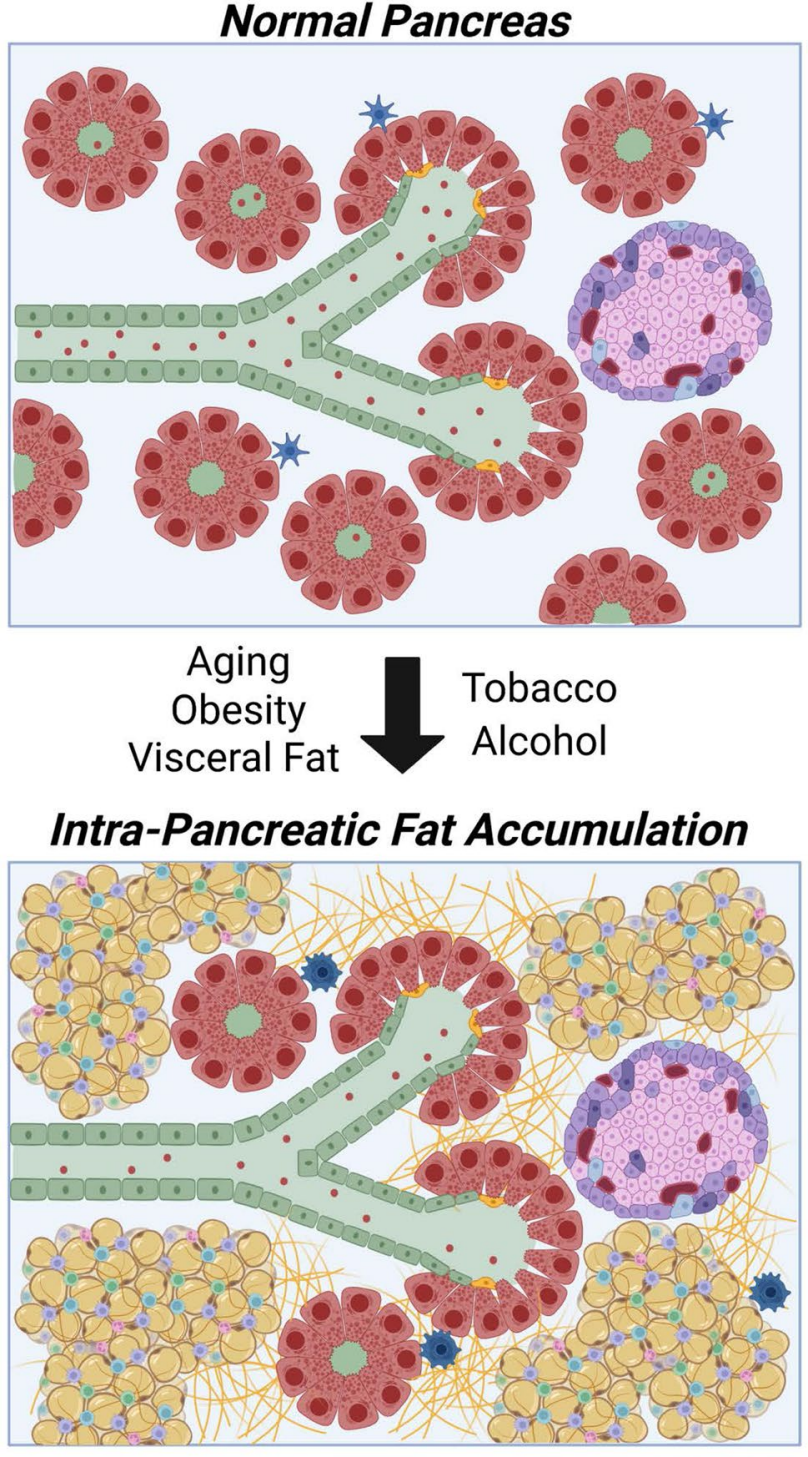
Accumulation of intrapancreatic fat. In response to increased aging, obesity, or visceral fat as well as tobacco or alcohol consumption, the pancreatic architecture is altered. Atrophy of exocrine pancreatic tissue leads to replacement with intrapancreatic fat cells, PSC activation and increased fibrosis; typically, islets remain intact. Created with BioRender.com

Obesity causes significant metabolic changes, which include changes in the hormonal landscape, and the over-abundance of various nutrients, including lipids. Excess lipids accumulate in lipid droplets, and in an increased population of enlarged adipocytes, in organs throughout the body. IPAs are repositories of ectopic fat that accumulate specifically in the pancreas [60, 61], and can gradually replace normal pancreas-resident populations such as acini, resulting in decreased exocrine pancreatic function [62–65] (Fig. 4). Large population studies have found the occurrence of IPAs is associated with increased age and body mass index (BMI) [65, 66]. Independent of age, IPAs are significantly elevated in obese individuals [67], particularly in those bearing elevated levels of visceral (rather than sub-cutaneous or thigh) fat [68, 69]. Other known and possible contributors to accumulation of IPAs include use of tobacco alcohol intake and low birth weight [70, 71]. IPA accumulation has been described as a risk factor for pancreatic diseases including acute [72] and chronic [73–75] pancreatitis, and pancreatic cancer [31, 59, 76–78]. Interestingly, extensive and rapid IPA accumulation has been observed in some individuals with hereditary conditions predisposing for pancreatitis. For instance, in infants in which loss of SPINK function has caused very rapid loss of acinar cells, there is widespread replacement of the acinar mass with adipocytes [79]. Similar substantial replacement of acinar tissue with adipose tissue has been reported in pediatric patients with inherited damaging variants in PRSS1 [80].

Lipid droplets (LDs) are intracellular organelles which exist in most cells to provide storage for fatty acids. In healthy individuals, a large part of the intracellular volume of adipocytes is composed of a large LD; however, the size and number of LDs resident in a given cell depends on nutrient availability, cell type, and pathological state. The structure of an LD consists of a core of neutral, uncharged lipids (with triglycerides most abundant, but also including cholesterol esters and minor lipid species) surrounded by a phospholipid monolayer which contains integrally and peripherally associated proteins that modulate LD function. The literature on pancreatic LDs is less developed than on IPAs. Obesity and excess nutrient availability have been associated with LD accumulation in endocrine and acinar cells. While a number of studies address the mechanisms of how LD accumulation results in altered endocrine cell function, pertinent to diabetes [81], studies of acinar cell LDs are typically observational, and often based on analysis of rodents fed high fat diets [82–84]; however, the abundance of evidence suggests increased LD levels in acinar cells is a common feature of obesity. Besides exocrine and endocrine tissue, PSCs also accumulate LDs containing vitamin A among other lipids; interestingly, these are lost as PSCs are activated to CAFs in the context of emerging PDACs [85–87]. To our knowledge, no studies have specifically investigated the relationship between LD accumulation in exocrine cells or PSCs and risk of pancreatitis or cancer.

### Mechanisms of pancreatic damage induced by excess IPAs and LDs

**Fig. 5:**
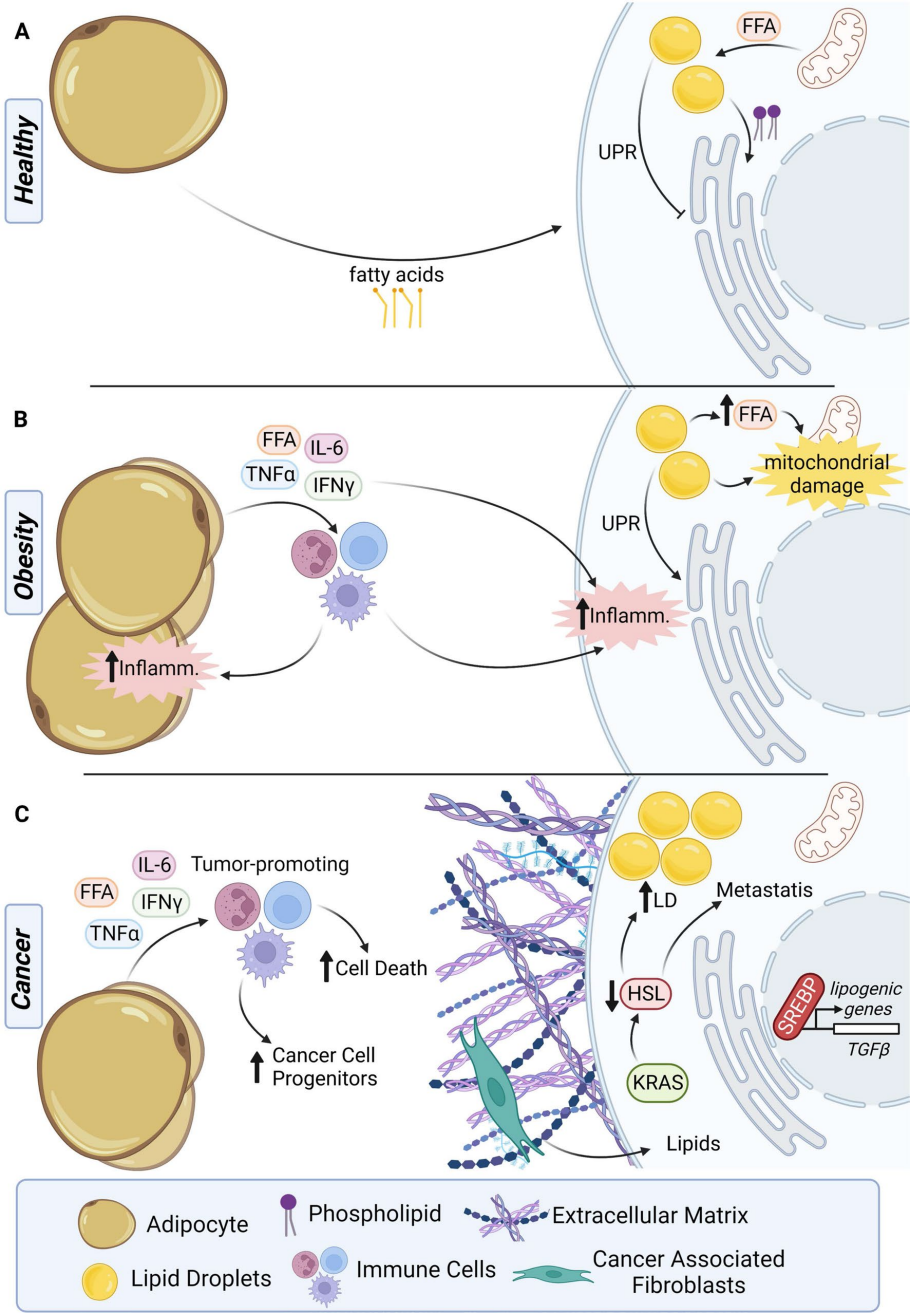
Adipocyte and lipid droplet dysregulation in pancreatic pathologies. **A** In healthy tissue, a limited number of adipocytes provide fatty acids to neighboring cells, provide a source of energy. Similarly, lipid droplets within healthy cells aid in balancing intracellular lipid levels, by sequestering free fatty acids (FFA) that would otherwise be oxidized to cytotoxic lipids. Lipid droplets also help to maintain phospholipid composition in the endoplasmic reticulum (ER), inhibiting the unfolded protein response (UPR). **B** In cases of obesity or high visceral fat, adipocytes secrete cytotoxic FFA as well as pro-inflammatory cytokines such as IL-6, TNFα, and IFNγ. These cytokines and FFA promote immune infiltration, which increase inflammation within adipocytes as well as cells. Similarly, cellular lipid droplets elevate intracellular cytotoxic FFAs, resulting in mitochondrial damage, and altering the phospholipid composition of the ER, promoting UPR. **C** In pancreatic cancer, the tumor microenvironment consists of a large amount of extracellular matrix (ECM), stroma, and cancer associated fibroblasts (CAFs) with a relatively small volume occupied by cancer cells. CAFs provide lipids to cancer cells to be used as building blocks for membranes and as an energy source. Within the cancer cells themselves, mutations activating the *KRAS* oncogene decrease expression of hormone-sensitive lipase (HSL), increasing the amount of lipid droplets in cancer cell as a method of promoting tumorigenesis. Decreased HSL and increased levels of SREBPs, transcription factors for lipogenic genes, are associated with increased metastasis. Besides promoting expression of genes involved in lipogenesis, SREBPs increase expression of growth factors such as *TGFβ* pancreatic ductal adenocarcinoma (PDAC) invasiveness and metastasis. Although PDAC has few to no adipocytes present in the area of established tumors, high levels of intrapancreatic adipocytes can help to promote a microenvironment advantageous to cancer formation, as release of pro-inflammatory cytokines and cytotoxic fatty acids promote infiltration of tumor-promoting immune cells that increase normal pancreatic cell death and increase presence of cancer cell progenitors. Created with BioRender.com

In healthy tissue, IPAs and LDs store lipids in readiness for metabolic demands, such as need to compensate for caloric restriction; under these conditions, lipolysis and lipophagy can process triacylglycerol to phospholipids and other required fatty acids. In cases of nutrient adequacy or surfeit, adipocytes and LDs possess additional protective functions, as recently reviewed [88] (Fig. 5). One important role is to protect cells from lipotoxicity associated with high levels of free fatty acids, which can cause cell damage via incorporation into membranes, or be processed via oxidation into cytotoxic lipids such as ceramide. LDs associate with the ER, among other cellular organelles; this is important for maintaining normal phospholipid composition of the ER membranes, and restraining induction of an unfolded protein response (UPR). LDs also protect mitochondria during autophagy, sequestering free fatty acids released during digestion of other cell compartments. In obese individuals, the storage capacity of LDs is overwhelmed, rendering tissue vulnerable to higher levels of free and cytotoxic fatty acids, induced UPR, and mitochondrial damage. This can be particularly deleterious in the context of additional factors that trigger cell and tissue stress, such as the hypoxia that characterizes PDAC and other tumor types [89].

Ectopic and visceral fat deposits, and in specifically increased abundance of adipocytes, have a well-characterized relationship with lipid-induced inflammation in tissues low in fat such as the liver and heart [20]. Adipocytes contribute to the development and progression of pancreatitis in several ways. Following damage to the pancreas (for instance, during ductal obstruction triggering acute pancreatitis), death of acinar cells causes release of digestive enzymes, including lipases, that cause necrosis of neighboring adipocytes. These release toxic levels of free fatty acids, and in particular unsaturated fatty acids, which reciprocally extend acinar injury [9, 25, 26, 90–94]. A recent retrospective patient study further cemented this connection between pancreatic fat and acute pancreatitis, finding there to be a significant association between IPAs and the occurrence of acute pancreatitis [95]. Acute pancreatitis has also been associated with defects in autophagy linked to mitochondrial dysfunction and impaired autophagy; both linked to obesity and accumulation of lipids [93]. In addition, the altered metabolism of adipocytes in obese individuals causes them to secrete a number of pro-inflammatory factors (IL-6, TNFα, IFNγ, MCP-1, IL-1β) that recruit activated macrophages and other immune cells to areas of tissue damage, and exacerbate necrotic responses [5].

Adiposity is associated with a tumor-promoting function for many types of cancer, based on both systemic and localized effects, delivered through multiple mechanisms [96]. In the case of pancreatic cancer, interpretation of the local role of IPAs is complicated by the significant changes in cellular composition that accompany tumor formation. Most PDACs are diagnosed at a late stage (3 or 4), at which point with the bulk of the solid tumor mass composed of CAFs and their secreted ECM, encompassing a minority population of cancer cells [97]. However, studies of very early stage tumors (e.g. [66, 77] and work generally analyzing the relationship between PDAC risk and adiposity have suggested a driver role of fatty pancreas in promoting early formation of pancreatic cancer. As with pancreatitis, the creation of an inflammatory microenvironment promotes the growth of oncogenically transformed cells, in part by contributing to the recruitment of tumor-promoting immune cells; it may also contribute by inducing localized cell death that expands the pool of proliferating incompletely differentiated acinar cell precursors, and other cancer progenitor cell populations.

One particularly intriguing recent study has also suggested that the size of LD pools within cancer cells is an important determinant of metastatic capacity [98]. In this work, the authors demonstrated that activated KRAS downregulated hormone-sensitive lipase (HSL), causing LDs to accumulate during the active growth phase of tumorigenesis. Subsequently, tumors undergo epithelial-mesenchymal transition (EMT) and other metabolic shifts as they become invasive; at this point, residual HSL allowed catabolization of LDs, promoting invasion and metastasis. A growing body of literature demonstrates that CAFs modulate PDAC growth by secreting lipids into the tumor microenvironment (e.g. [99].). In addition, PDAC tumors reprogram their fatty acid and cholesterol biosynthesis pathways to maximize tumor growth and invasion, with some evidence that differences between PDACs in pyruvate flux and the balance between glycolysis and lipogenesis influencing tumor aggressiveness [87, 100–102]. Activity of SREBP, a core transcription factor promoting genes required for lipogenesis, has been linked to specification of PDAC subtype, with reduction in available cholesterol inducing SREBP, which in turn triggers production of TGFβ, and promoted tumors with features of the basal subclass [101]. In a number of tumor types, SREBP activity has been shown to be enhanced by interactions with MYC [103]. Given MYC is induced during expansion of acinar cell populations, which accompanies recovery from pancreatic injury, MYC-SREBP signaling may represent an alternative approach to triggering lipogenesis. This reprogramming is likely modulated by IPAs and abundance of LDs, but detailed mechanisms remain to be established.

### IPAs in mouse models

There has been extensive description of the clinical presentation and imaging of intrapancreatic fat in human patients [19, 78, 104, 105]. There is evidence that IPAs can accumulate through at least two routes; fatty replacement, potentially by transdifferentiation of pancreatic-resident cell populations, following induced death of acinar cells, and fatty infiltration of adipocyte precursors from other tissues [106]. However, the specific cell of origin for IPAs and signaling defects triggering IPA accumulation, and the relationship of different inducers of IPA accumulation to PDAC risk and presentation are not currently well defined, with suggestions that IPAs may arise from distinct causes as a result of acute or chronic pancreatitis, obesity, diabetes, or other pathogeneic stimuli. Few studies have taken the induction of IPAs as their primary focus. However, over the past two decades, studies using dietary and genetic manipulation of mouse models have provided unexpected insights into triggering factors for acinar cell loss and IPA accumulation. This work has identified a surprisingly diverse set of conditions with these properties, as summarized below (Table 1).

**Table 1:** Mouse models resulting in loss of acinar cells and increased intrapancreatic adipocytes

Mouse models		Effect			Ref
Dietary	High fat diet			Adipocyte hypertrophy, inflammation	[107, 108]
Pancreatitis	Blocked pancreatic duct			Acinar cell death and replacement by adipocytes	[110, 111]
	Constitutively active trypsin			Desmoplasia and adipocytes replace acinar cells	[112]
Genetic	Whole body	*Arf4*	Knockout	Acinar cell loss accompanied by fibrosis and adipocyte replacement, unaffected endocrine cells	[165]
		*Cby1*	Knockout	Loss of cilia, decreased hedgehog signaling, pancreatic atrophy, adipocyte replacement	[156]
		*Ift88*	Knockout	Acinar loss, increased ducts and fatty replacement, unaffected endocrine cells	[154, 155]
		*Pdx1*	Knockout	Loss of acinar cell mass and replacement by adipocytes	[124]
		*Postn*	Knockout	Pancreatitis induced acinar-to-adipocyte trans-differentiation	[113]
		*Prox1*	Knockout	Defects in pancreatic morphogenesis	[121]
		*Snail1*	Knockout	Acinar replacement with adipocytes, unaffected endocrine cells	[139]
		*TgfbrII*	Overexpression	Increased duct accumulation, ADM, fibrosis, fatty replacement	[170]
		*Ubaid1*	Knockout	Oxidative stress, acinar cell apoptosis, adipocyte replacement	[169]
	Total pancreas	*Apc*	Knockout	Pancreatic hypertrophy	[132]
		*c-Myc*	Knockout	Acinar cell loss from embryogenesis, acinar-to-adipocyte trans-differentiation	[133]
		*Hnf6*	Knockout	Duct dilation, ADM, acinar cell loss, adipocyte accumulation, fibrosis, some adipocyte trans-differentiation	[158]
		*Ikkα*	Knockout	Fibrosis, ductal metaplasia, acinar atrophy, fatty replacement	[168]
		*Jag1*	Knockout	Reduced ductal ciliation, reduced acinar cell tissue, fibrosis, replaced adipocytes	[163]
		*Kif3a*	Knockout	Acinar loss, increased ducts and fatty replacement, unaffected endocrine cells	[152]
		*Prox1*	Knockout	Defects in acinar and ductal cell lineage, fatty replacement of acini	[120]
		*Tgfβr1*	Overexpression	Acinar loss, ADM, accumulation of adipocytes	[171]
	Acinar	*c-Myc*	Overexpression	Defects in acinar differentiation	[134]
		*Gata6*	Knockout	ADM, adipocyte infiltration, mucinous metaplasia	[128]
		*Hes1*	Knockout	Pancreatic atrophy, fatty replacement, immature acinar cells with reduced replication	[163]
		*Tgfβr1*	Overexpression	Acinar loss, ADM, accumulation of adipocytes	[171]
	Ductal	*Hnf1β*	Knockout	Decreased genes related to ciliary formation, loss of cilia and ducts, acinar cell apoptosis, ADM, adipocyte infiltration	[160]
		*Hnf6*	Knockout	Duct dilation, ADM, acinar cell loss, adipocyte accumulation, fibrosis	[157, 159]
		*Lkb1*	Knockout	Duct dilation, ADM, acinar cell loss, adipocyte accumulation, fibrosis, some adipocyte trans-differentiation	[158]

*Dietary models*. Studies seeking to simulate NAFPD in mice typically are based on comparison of wild type mice fed either a standard or high fat diet (HFD) (10% versus 60% fat content) for 8 or more weeks. In two early studies [107, 108], these regimens resulted in increased body mass, greater visceral fat, and overall adipocyte hypertrophy. The HFD induced multiple indicators of pancreatic pathology, including accumulation of intracellular lipid droplets within pancreatic acinar cells, adipose infiltration, and intralobular fat. These were accompanied by increased fibrosis, increased infiltration of M1 and M2 macrophages (indicative of inflammation), glucose intolerance, insulin resistance, and morphological changes in alpha and beta cells. Together with other changes affecting the liver and additional organs induced by a HFD, this approach more closely simulates the pathologies associated with human obesity than use of genetic models such as *ob/ob* mice, although these have also been used to document a relationship between HFD and accumulation of pancreatic adipocytes [109]. In these latter studies, pancreata from *ob/ob* animals fed HFDs had elevated levels of cholesterol and triglycerides, free fatty acids (including saturated fatty acids), and the pro-inflammatory cytokines TNF-α and IL-1β versus wild type (wt) mice on the same HFDs [109].

*Induced acute and chronic pancreatitis*. Blockage of the main pancreatic duct—a common cause of acute pancreatitis—has been shown in mouse models to cause extensive rapid death of acinar cells. In studies using this model, the initial phenotype which simulates acute pancreatitis, progress over a month to resemble chronic pancreatitis, with evidence of fibrosis, ductal dilation, and extensive inflammatory cell infiltration. In two independent studies, ductal blockage led to replacement of acinar cells with extensive adipose tissue [110, 111]. In one study, it was proposed that an undefined population of mesenchymal cells within the pancreas may have differentiated into IPAs [111]. An alternative strategy by introduction of a D23A constitutively active allele of trypsin has been shown as a physiologically relevant model of chronic pancreatitis in which release of activated trypsin from acinar cells resembles the mechanism of the human genetic predisposing syndromes [112]. These mice developed spontaneous acute, then chronic pancreatitis, and a large-scale replacement of acinar cells with desmoplasia and adipose tissue. Interestingly, though acinar cells and much of the exocrine pancreas deteriorated, Islet of Langerhans remained largely intact and functional.

In contrast, despite the widespread use of cerulein treatment as a means of inducing acute or chronic pancreatitis, there are no reports suggesting cerulein induction of pancreatitis impacts the accumulation of IPAs in the short term (e.g. [113]). Whether this reflects a biological difference associated with blockage versus chemically-induced ablation, or whether this topic has not yet been investigated, requires study.

*Genetic Models*. To date, there has been little if any use of genetic models to specifically probe regulation of adipocyte accumulation in the pancreas. However, a surprising number of studies that used genetic knockouts to investigate pancreatic development or various pancreatic pathologies have serendipitously identified genes and pathways influencing accumulation of intrapancreatic adipocytes. While in most cases the specific cell populations that are the source of pancreatic adipocytes were not investigated, some consistent themes emerge from the studies summarized here.

**Fig. 6:**
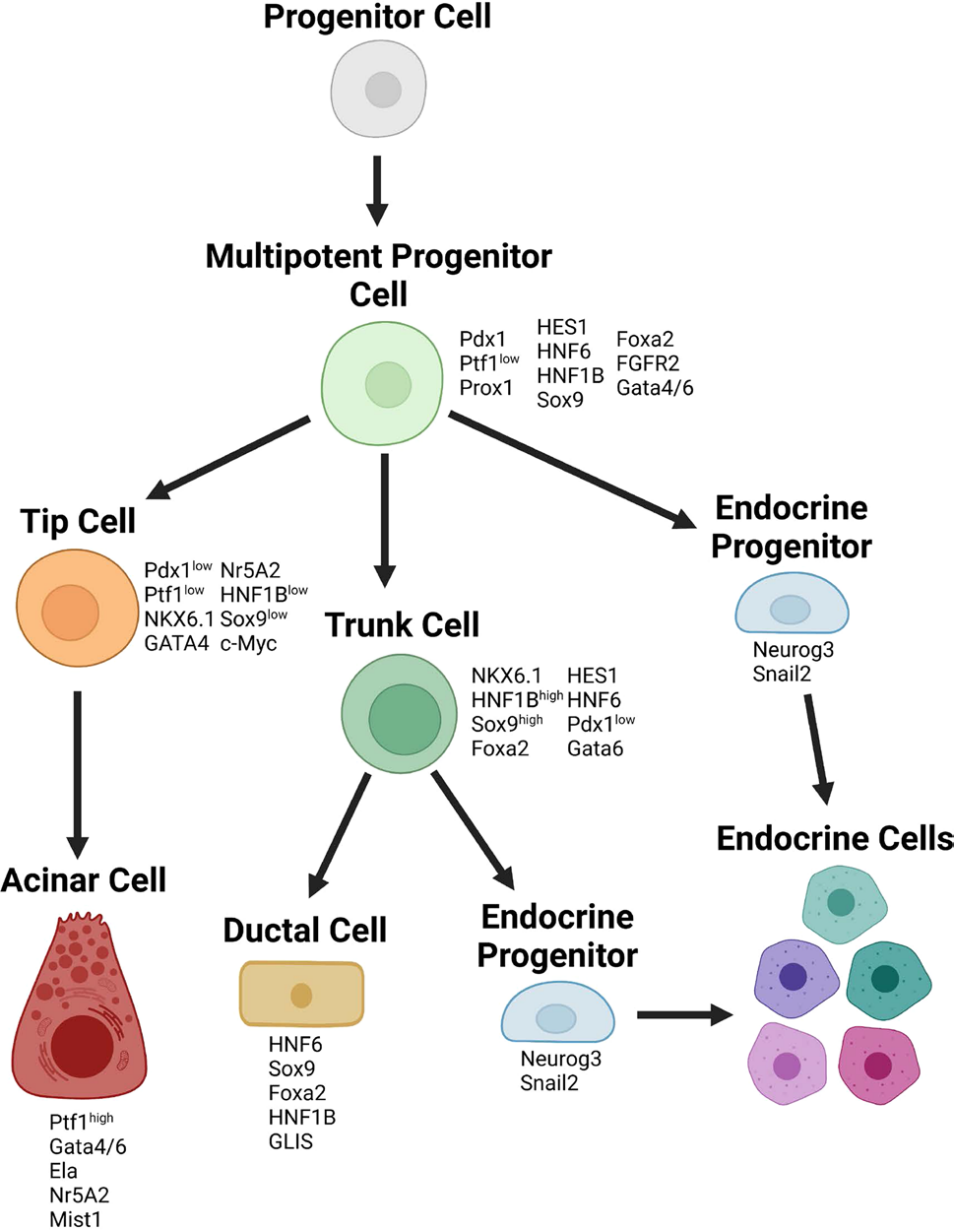
Pancreatic cell fate. During embryonic development, the pancreas arises from an endoderm progenitor cell [191]. This initial progenitor cell differentiates into a pancreatic multipotent progenitor cell capable of differentiating into any endocrine or exocrine pancreatic cell lineage, excluding PSCs. From multipotent progenitor cells, tip, trunk, and endocrine progenitor cells arise, each with differing cell lineage markers. Tip cells terminally differentiate into acinar cells. Trunk cells differentiate into ductal cells and endocrine progenitor cells. Both endocrine progenitor pools differentiate into the pool of endocrine cells that make up Islets. Each progenitor and terminally differentiated cell type is specified by a set of unique transcription factors associated with cell lineage. Created with BioRender.com

*Context: normal pancreatic development*. It is important to place phenotypes emerging from the genetic models in the context of the normal roles of disrupted genes in pancreatic morphogenesis and maintenance. Formation of the pancreas depends on the orchestrated activation and inhibition of a number of distinct transcription factors that specify pancreatic fate and establish cell lineage (Fig. 6) beginning at embryonal day E8.5, as detailed in numerous reviews [114–117]. These include master regulators such as PDX1, which establish overall pancreatic identity, other factors critical for maintaining pancreatic lineage differentiation in expanding pools of progenitor cells during normal development (PTF1, SOX9, HES1, FGFR2, NKX6-1, PROX1, HNF6, HNF1b, FOXA2, GATA4 and GATA6). These proteins cross-signal to each other, and maintain multipotent precursor pools in the pancreatic bud. Days E11-E15 are marked with a process of branching morphogenesis during which acinar cells emerge from the “tips” of growing pancreatic bud branches whereas ductal and endocrine cells emerge from cells localized closer to the trunk of the branching “tree”. This process is associated with segregated expression and activity of transcription factors. For example, by day E13.5, PTF1 is solely expressed in acinar cells at the tips, whereas the trunk region expresses NKX6, HNF1B, SOX9, HES1, and HNF6, and produces bi-potential precursors to ductal and endocrine cells. In late embryogenesis, precursor cells with bi-potency are lost, with emerging unipotent precursors for ductal cells expressing HNF6, SOX9, and GLIS3, acinar precursors expressing PTF1, MIST, and HES1, and endocrine precursors expressing NEUROG3 among other transcription factors. Each of these specify a further downstream program that results in terminal differentiation of mature ductal cells, and various lineages of endocrine cells. As acinar, ductal, and endocrine cell populations mature, they acquire additional cell-type specific signaling features appropriate for their physiological roles, while relinquishing others. For example, activity of the pro-proliferative factor c-MYC is specifically relevant to the maintenance of acinar cell mass, which represents 90% of the mature pancreas, and is induced during the rapid tissue regeneration following pancreatic damage [117, 118]. Subpopulations of acinar cells, called centro-acinar cells, retain progenitor-like transcripts that enable self-renewal and rapid regeneration of the exocrine compartment following acute pancreatitis [119].

**Fig. 7:**
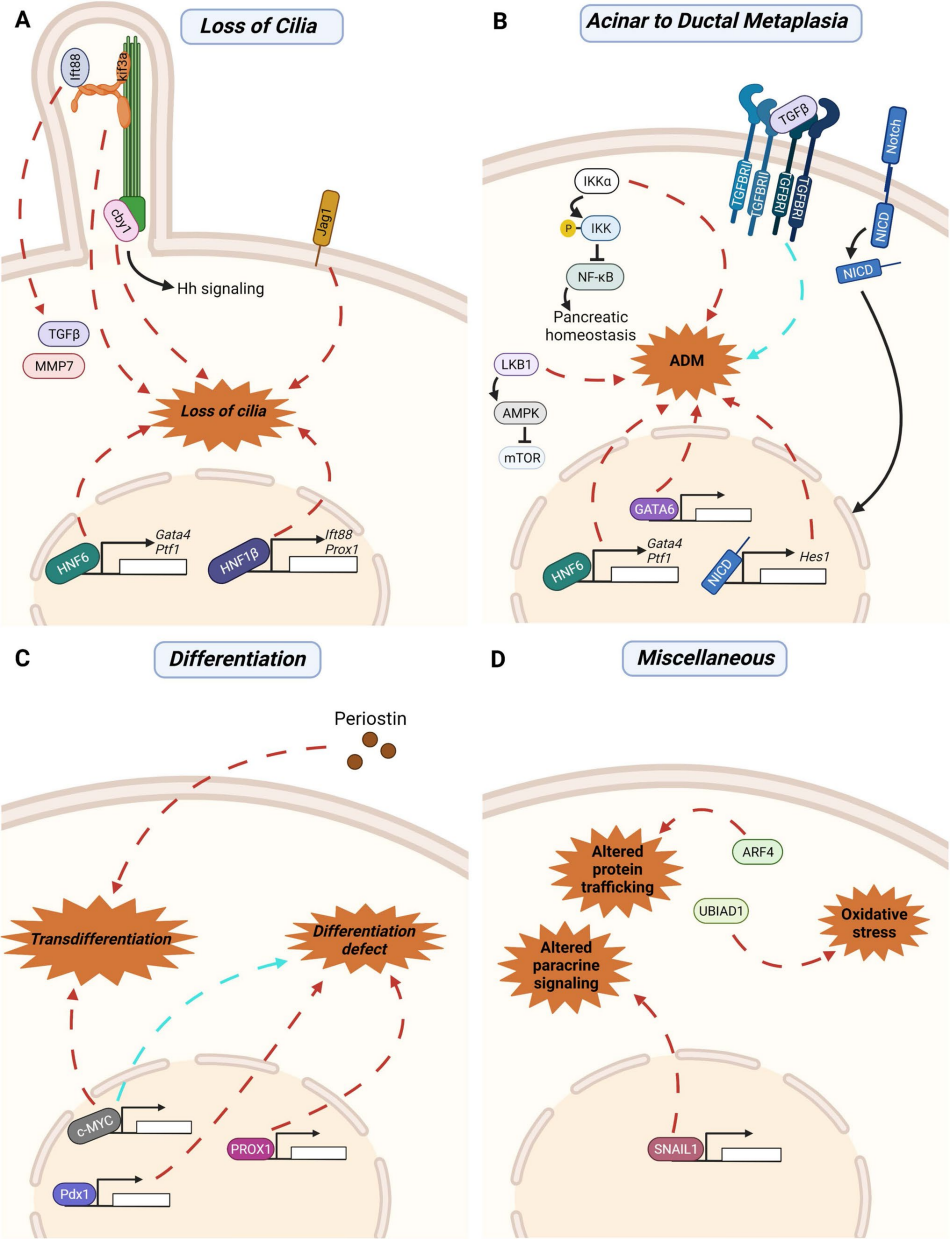
Signaling that promotes acinar atrophy and pancreatic adiposity. **A** Knockout (indicated by red arrows) of ciliary genes (*Kif3a*, *Ift88*, *Cby1*), transcription factors (*Hnf6*, *Hnf1β*), and Notch signaling pathway genes (*Jag1*) result in a loss of cilia within ducts, accompanied by increased pancreatic fibrosis (mediated by TGFβ and MMP7), acinar cell atrophy, and intrapancreatic adipocyte infiltration. **B** Knockout of *IKKα* (a regulator of NF-κB) and *LKB1* (a regulator of mTOR) both result in increased rates of Acinar-to-Ductal metaplasia (ADM). Knockout of genes encoding transcription factors implicated in pancreatic differentiation (HNF6 and GATA6) and *Hes1* (a target of Notch signaling) also increase rates of ADM. Lastly, increased activity of TGFβ via overexpressed (indicated by blue arrows) TGFRII and TGFRI also increased rate of ADM within the pancreas. In all of these cases, increased rates of ADM were associated with acinar cell loss and adipocyte replacement. **C** Genetic manipulations that targeted factors involved in acinar cell differentiation resulted in pancreatic adipocytes were. Loss of transcription factors *Pdx1* and *Prox1*, as well as overexpression of *c-Myc* resulted in a defect in pancreatic morphogenesis and impaired differentiation of mature acinar cells, causing extensive adipocyte infiltration. Conversely, loss of pancreas-wide expression of *c-Myc* also resulted in a loss of acinar cells due to trans-differentiation of acinar cells to adipocytes. Knockout of *periostin* (*Postn*), a protein secreted by activated PSCs, also resulted in acinar-to-adipocyte trans-differentiation. **D** Loss of *Arf4*, a ciliary gene, causes altered protein trafficking, potentially influencing acinar cell capacity to secrete digestive enzymes. Loss of *Ubiad1*, a gene encoding an enzyme involved in vitamin K synthesis, results in increased oxidative stress. Lastly, knockout of *Snail* expression alters the secretome of mesenchymal cells, altering paracrine signaling within the pancreas. In each of these models, the loss *Arf4, Ubiad1,* or *Snail* causes exocrine atrophy accompanied by increased pancreatic adiposity. Created with BioRender.com

*IPA accumulation following misexpression of factors regulating pancreatic morphogenesis*. A large body of data from use of mouse knockout or transgenic models implicate a number of genes in pancreatic morphogenesis. The below examples (results summarized in Fig. 7) emphasize some common points: first, that a predominant phenotype arising from disruption of pancreatic morphogenesis is depletion of the acinar cell compartment, with particular loss of mature acinar cells; and second, that a frequent consequence of these disruptions is accumulation of adipocytes, which replace the acinar cells. Notably, while in some cases defects in pancreas morphology are detected in embryos, in other cases pancreata appear morphologically normal at birth, with defects only appearing after several weeks.

*Prox1.* The homeobox-containing transcription factor PROX1 is expressed prior to the emergence of the pancreatic bud, and is expressed in multipotent progenitor populations of the pancreas. Upon differentiation to multiple lineages, levels of PROX1 are maintained in centroacinar, ductal and islet cells, but become nearly undetectable in acinar cells. Studies of *Prox1* deletion using the early *Pdx1-Cre* knockout system [120] or in Prox1 null mice [121] in each case revealed defects in pancreatic morphogenesis affecting multiple cell lineages, beginning as early as embryonal day E13.5. Defects were observed in the ductal cell lineage, and included increased ductal cell proliferation and altered duct morphogenesis. A complex sequence of acinar cell defects was also observed. Premature differentiation to mature acinar cells occurred in embryos, but this was compensated for by time of birth, so that acinar cells occurred in normal numbers and appeared morphologically normal. However, in the postnatal period, there was progressive and extensive loss of acinar cells, which were replaced by adipocytes in adult mice. In addition, the pancreata of older *Prox1*-deficient mice developed signs of pancreatitis, including fibrosis, acinar cell apoptosis, and infiltration of macrophages and neutrophils. Genetic lineage tracing using a β-galactosidase marker showed the origin of adipocytes as distinctly separate from the epithelial lineage in the mice with Pdx1-Cre-driven deletion of *Prox1*. In 3-month-old mice, acinar cells were positive for β-galactosidase, but adipocytes were not, suggesting acinar-to-adipocyte differentiation was not the mechanism for adipocyte infiltration. This raises a provocative question as to whether there is an *ex vacuo* growth of adipocytes, or if the IPA population expands due to insulin-driven signaling.

*Pdx1*. The homeodomain transcription factor *Pdx1* is initially expressed in the gut, specifying the region giving rise to the pancreas primordium. As the pancreas forms, around day E8.5–9.5 *Pdx1* is initially expressed in all cellular compartments [114–117], but later (day 15) is restricted to the endocrine lineage. Loss of *Pdx1* in mouse models [122] or humans [123] causes pancreatic agenesis. In contrast, in transgenic mice with forced continuous expression of *Pdx1* in exocrine cells, mice were born with normal pancreata [124]. However, by the time mice were ~ 3 weeks of age, exocrine areas had gaps, with progressive loss of acinar cell mass. By 8 weeks of age, 30–40% of the pancreatic mass was composed of adipocytes, accompanied by a large increase in pancreatic triglyceride levels. As the animals aged, acinar cells became increasingly disorganized with evidence of enhanced replication and apoptosis, and reduced production of digestive enzymes including amylase [124].

*Gata4 and Gata6*. The GATA4 and GATA6 transcription factors are expressed early throughout the pancreatic primordium. With largely overlapping function, loss of both is associated with pancreatic agenesis [125, 126]. As development progresses, after day ~ E16.5, expression of GATA6 is restricted to ducts and endocrine cells [127]. Martinelli and colleagues used *Gata6*^*loxP/loxP*^*;Ptf1a-Cre*^+*/KI*^ mice to knock out *Gata6* in pancreatic epithelial precursors beginning from embryonal day ~ 9 [128]. While pancreata were normal at birth, morphological abnormalities including focal ADM and appearance of adipocytes were noted by the time mice were 1 month old; at this time, acinar cells had higher rates of both proliferation and apoptosis than controls. Signs of accumulation of lipid droplets within acinar cells and amylase within adipocytes suggested trans-differentiation based on epithelial lineage tracing with an EYFP marker in some adipocytes and an extensive upregulation of the pro-adipogenic proteins PPARγ and perilipin in acinar cells. By 7 months old, acinar cells were nearly absent, almost completely replaced by adipocytes, with signs of ADM and mucinous metaplasia [128].

*c-Myc: required for acinar expansion*. Interest in study of c-Myc arose following earlier studies investigating the role of the WNT-APC-CTNNB1 signaling axis in pancreatic differentiation. In this work, deletion of the CTNNB1 transcription factor resulted in specific loss of the exocrine pancreas, with some studies reporting signs of ADM [129–131]. Complementary experiments analyzing mice with pancreas-specific deletion of *Apc*, a negative regulator of CTNNB1, showed a reciprocal phenotype of pancreatic hypertrophy, driven by overgrowth of acinar cells. This hyperplasia was reversed by deletion of *c-Myc* in *Apc*-deficient pancreata [132]. C-MYC is a CTNN1-dependent transcript, encoding a transcription factor that promotes progenitor cell proliferation, and specifically supports the large expansion of acinar cell precursors in pancreatic development [117].

Pancreatic inactivation of floxed *c-Myc* either with *Pdx1-Cre* (all pancreatic epithelial precursors, [133]) or *Ptf1-Cre* (acinar lineage precursors, [134]) in each case caused loss of acinar cell mass detectable by late embryogenesis or time of birth. The more extensive study by Bonal et al*.* established that loss of *c-Myc* caused a progressive loss of acinar mass, and progressive accumulation of adipocytes, with these comprising the bulk of the pancreatic mass in adults [133]. Analysis of the expression of factors associated with pancreatic differentiation indicated downregulation of PTF1 and MIST1, required for specification of acinar cells, by the time of birth. Notably, this study identified transitional cells with features of both acinar cells and adipocytes, including acinar cells containing small lipid droplets, and cells with extensive fat and retaining zymogen granules, but lacking some expression of PTF1 and MIST1. In addition, lineage tracing experiments using a *Pdx1-Cre* transgene to irreversibly activate the expression of the reporter R26R or R26-EYFP in parallel with inactivation of c-MYC mice showed activation of this reporter in the cytoplasm of pancreatic adipocytes by the time mice were two months old, suggesting direct acinar-to-adipocyte trans-differentiation [133]. Notably, lineage tracing revealed similar acinar origins of accumulated adipocytes in the pancreata of aged mice, or following cerulein treatment and pancreatic regeneration. Intriguingly similar transitional cells, with acinar-appearing cells containing lipid droplets and adipocytes with residual granules, were found in an aged human pancreas; however, only one pancreas was analyzed in this study [133].

Complicating the understanding of c-MYC function in acinar maturation and differentiation controls, a subsequent study used c-MYC overexpression under the control of the Ela1 (elastase) acinar-specific promoter [135]. This study found that overexpression of c-MYC in acinar precursors also resulted in profound defects in differentiation programs, and elevated rates of both proliferation and apoptosis. c-MYC negatively regulated PTF1a activity by direct binding to PTF1a and repressing its transcriptional activity, and impairing the ability of PTF1a to bind the promoters of digestive enzymes. While early differentiation markers of acinar cells were retained and pre-acinar cells were retained in the pancreas, acinar maturation was blocked, and a “liver-like” transcriptional signature similar to that previously reported for knockouts of the PTF1a complex component RBPJL [136] or elevation of glucocorticoid signaling [137] was detected. Interestingly, this study found c-MYC overexpression was a characteristic of transformation by *KRAS*, and found in PanINs [135]. Together with the earlier work, this study suggested downregulation but not complete absence of c-MYC was required for normal differentiation of acinar cells, and that pre-acinar cells could adapt more than one abnormal fate based on anomalously low or high c-MYC levels.

*Genes expressed in mesenchymal cells: SNAIL and POSTN*. Some genes causing pronounced replacement of acinar cells with adipocytes are not expressed in epithelial cells. The SNAIL1 transcription factor is typically expressed in mesenchymal cells, and represses genes associated with a differentiated epithelial identity. SNAIL plays an essential role in embryogenesis, supporting cell migration and pattern formation, but has minimal expression in healthy adult tissues [138]. Tamoxifen-induced loss of *Snail1* in all tissues under control of β-actin-Cre-ER of adult mice caused rapid loss specifically of pancreatic mass and function, and rapid replacement of acinar cells with adipocytes, which comprised nearly half the volume of the pancreas; islets were unaffected [139]. In contrast, ablation of *Snail1* using the *Ptf1* promoter to express tamoxifen-regulated Cre specifically in adult acinar cells had no effect. Further analysis confirmed activity of SNAIL1 was selective to CD105-positive mesenchymal cells, and that loss of *Snail1* significantly altered the secretome of the mesenchymal cells, suggesting a paracrine effect [139]. Induction of MYC in acinar cells using an elastase 1 c-Myc (*Ela-1-MYC)* transgene typically gives rise to acinar carcinomas, as well as tumors that display both acinar-like and duct-like features [140]. In the context of *Snail1* deficiency, although tumors still formed in *Ela-1-MYC mice*, the tumors were predominantly of ductal morphology, suggesting an effect on lineage control [139].

As another example, the ECM protein periostin (POSTN), known to promote tumorigenesis [141], is secreted by activated pancreatic stellate cells and is highly upregulated in the stroma in cases of chronic pancreatitis and PDAC [113]. In mice with total body *Postn* knockout, no disruption of normal pancreatic development was apparent. However, response to administration of cerulein to induce acute pancreatitis was significantly altered, with acinar cell replacement by adipocytes noticeable by 7 days after treatment, and at 3 week causing adipocytes to replace nearly the entire mass of the pancreas [113]. This change was attributed to acinar-to-adipocyte trans-differentiation, based on downregulation of the transcription factors *Mist1* and *Rbpjl*, which promote acinar identity and maturation [142], upregulation of PPARγ, which promotes adipocyte identity, and detection of amylase-containing granules in the cytoplasm of adipocytes [113].

*A role for loss of ciliary integrity in pancreatic adipocyte formation*. The primary cilium is an organelle that has been described as a cellular “antenna”. In many lineages of non-cycling cells, a cilium is formed from the mother centriole, which localizes proximal to the plasma membrane and extends a central microtubule-based axoneme encased with a specialized membrane into the extracellular environment [143]. This specialized structure is the locale for receptors for a number of growth factors; in addition, for cells lining ducts, cilia commonly protrude from the apical cell surface into the duct, where they act as flow sensors, regulating the activity of downstream signals that regulate cell growth and differentiation. defects in cilia define a group of pathological conditions termed ciliopathies, many of which are associated with obesity [144]. Conditional loss of *Kif3a* or *Ift88* (genes required for ciliary formation) in all tissues leads to increased adiposity and hepatic steatosis [145]. While there is considerable evidence for local action of cilia in affected tissues, interpretation of the mechanism of ciliary loss in whole body ablation studies is complicated by evidence that loss of cilia contributes to hyperphagia, likely due to a role in hypothalamic neurons which govern appetite [146]. However, several studies specifically addressing cilia expressed on pre-adipocytes or mesenchymal stem cells (MSCs) have linked variation in ciliary length, and accompanying availability of ciliary receptors for WNT, IGF-1 and Hedgehog (HH), to the transition between a pre-adipocyte to commitment to adipocyte differentiation [146–148]. Although not studied in the setting of the pancreas, it is also of interest that ciliation has also been shown to influence adipogenesis in injured muscle tissue [149] and in mesenchymal precursor cells [150], based on altered response to HH and other signaling factors.

In the pancreas, ductal and endocrine cells express cilia, while most acinar cells do not. Intriguingly, a number of studies have linked loss of cilia or ciliary defects on ductal cells with pancreatitis. One early study found that multiple rodent models of pancreatitis (alcoholic, ischemic, and obstructive) caused a reactive increase in ciliary length, which the authors proposed was a “reaction of defensive factors against the increase of pancreatic duct pressure” [151]. Conversely, several genetic studies have demonstrated that ciliary defects in pancreatic ductal cells causes pancreatitis and replacement of pancreatic tissue with adipocytes.

One of the first genetic studies to identify unexpected pancreatic infiltration used a variant of the Pdx1 promoter, expressed from ~ embryonal day 10.5 in all epithelial cells of the developing pancreas, to drive Cre-flox dependent ablation of *Kif3a* [152]. Kif3a is a subunit of kinesin-2, an anterograde motor which is essential for intraflagellar transport within the cilium; in the absence of Kif3a, cilia do not form, and cells are non-responsive to potent morphogenic growth factors such as Hh [153]. This study found loss of Kif3a in the embryonic pancreas resulted in loss of acinar cells and expansion of the ductal compartment by the time mice were two weeks of age, accompanied by enhanced expression of the pro-fibrogenic factor TGFβ, the matrix metalloprotease MMP7, and increased fibrosis. Notably, in 2 month old mice, large areas of the pancreas were replaced by adipose tissue [152]. Similar findings emerged from two studies of loss of *Ift88* (also required for intraflagellar transport and ciliation) [154, 155], with disorganization of acinar structure and acinar loss in the early post-natal period, expansion of pancreatic ducts, fibrosis, and extensive appearance of adipose cells beginning by ~ 3 weeks of age, but little evidence of effect on endocrine cells. In one of these studies, there was evidence for premature activation of CPA in acinar cells, suggesting autodigestion may contribute to loss of acini [155]. Another study investigated the role of Chibby (*Cby1*), a protein localized to the ciliary base and essential for ciliary formation [156]. Constitutive knockout of *Cby1* caused loss of cilia and diminished Hh signaling, with extensive pancreatic atrophy and replacement with adipocytes commencing with a few days of birth and accompanied by dilation of ducts, fibrosis, and immune cell infiltration. Although Cby1 protein was not detected on acinar cells, a single cell sequencing analysis accompanying this study identified a sub-population of acinar cells expressing Cby1, although the identity of this population and whether it was transitional (to ducts or adipocytes) was not pursued. However, detailed examination indicated a secretion defect and zymogen granule accumulation in acinar cells of Cby1 knockout mice, in line with a model of autodigestion [156]. In all of these studies, the cellular precursor for these infiltrating adipocytes was not investigated.

*Transcription factors controlling ductal differentiation, cilia, and adipocyte accumulation*. In differentiation of the normal pancreas, transcription factors active in multipotential precursor cells (MPCs) activate downstream effectors that specify distinct cell lineages. Hnf1β, active in MPCs, induces expression of the transcription factor Hnf6/Onecut (OC1), which in turn transcriptionally activates genes required for acinar and ductal lineages (including *Gata4, Ptf1, Nr5a2*). Some of these transcription factors retain activity in ductal cells in the adult, contributing to maintenance of cell identity. Several studies have investigated the consequence of induced loss of *Hnf1*β or *Hnf6* at various points in development of the pancreas [157–160].

A thorough study used a *Sox9-CreER;Hnf1b*^*fl/fl*^*;R26RYFP* construct to specifically induce loss of *Hnf1β* in ductal cells either at postnatal day 3, or in adults. Loss of Hnf1β in neonates surprisingly led to retention of expression of genes required for ductal differentiation (*Sox9, Hnf6, Cftr*), but significantly downregulated *Ift88*, *Prox1*, and other genes required for ciliary formation and ductal architecture, and caused loss of cilia in ducts, and expansion of ductal cell area [160]. Within a week, there was notable loss of acinar cells and signs of acinar cell apoptosis, and within a month, there were signs of ADM, extensive infiltration of adipocytes, and fibrosis; the pancreata of adult mice were nearly full of adipocytes, and mice experienced chronic pancreatitis and weight loss most likely due to exocrine deficiency. Notably, many mice with postnatal loss of *Hnf1β* developed spontaneous early PanINs; although these did not progress, mice deficient in *Hnf1β* developed accelerated formation of high grade PanINs when combined with a KRAS mutation [160]. Deletion of *Hnf1β* in adult mice removed the ability of the mice to recover from cerulein treatment, leading to rapid onset of chronic pancreatitis that also progressed towards PanIN formation [160].

*Hnf6* was deleted using a variety of strategies, including use of Sox9-CreER transgene to induce loss in ductal cells between days P2-P10 [158], or under control of Pdx1-Cre, to induce loss in pancreatic MPCs [157, 159]. These studies revealed similar pancreatitis phenotypes, including ductal dilation within 3 weeks of age, and progressive acquisition of phenotypes including loss of cilia on ductal cells, acinar cell loss, ADM, very extensive adipocyte accumulation, immune cell infiltration, and fibrosis. Notably, lineage tracing experiments in one study suggested that a minority population of adipocytes were transdifferentiated from acinar cells, but suggested that most were not [158].

*Notch signaling: Jag1 and Hes1*. The Notch signaling pathway, also linked to ciliary function in some cases [161, 162], is required for pancreatic development, with disruption of the Notch activating ligand Jagged (Jag1) associated with Alagille syndrome in humans, characterized by exocrine insufficiency. Knockout of *Jag1* under the control of the *Pdx1* promoter in mice caused mice to be born with a relatively normal pancreas. However, by 6 weeks of age, ducts are enlarged and ciliation of ductal cells reduced, the ductal tree disrupted, and acinar tissue extensively reduced and fibrotic, and replaced by adipocytes, in a phenotype that continued to progress [163]. Lineage tracing experiments excluded the possibility of adipocytes differentiating from pancreatic acinar cells, suggesting a likely origin from transdifferentiated mesenchymal cells [163].

The transcription factor *Hes1* is an important downstream target of Notch signaling [164]. In embryonic development, *Hes1* drives cells towards a ductal phenotype; in adult pancreata, expression of Hes1 in ducts and centroacinar cells prevents these cells from gaining acinar features, while maintaining a progenitor pool. Hes1 is induced following acute pancreatic damage from cerulein treatment, and required for regeneration of the exocrine compartment. In mice with induced loss of Hes1 under control of the *Ptf1* (acinar-cell restricted) promoter, mice were born with normal pancreata, but by 12 weeks of age, there was extensive pancreatic atrophy, and replacement with adipose tissue. Residual acinar cells had elevated expression of markers of immature acinar cells, and showed reduced rates of replication. Following cerulein treatment, regeneration was impaired, with a high rate of ADM observed. Surprisingly, in the context of an activated KRAS transgene, *Hes1* loss was associated with fewer PanINs, but a faster rate of PDAC formation [163].

*LKB1*. Liver Kinase B1/SerineThreonine Kinase 11 (Lkb1/Stk11) is an upstream positive regulator of AMPK and other related kinases which regulate cell growth, metabolism, and polarity. Of relevance to obesity, LKB1/AMPK negatively regulate mTOR, serving as a metabolic checkpoint; in cases of chronic high blood sugars and lipids, LKB1/AMPK signaling is depressed [35]. Knockout of Lkb1 using the Sox9-CreER transgene to induce loss in ductal cells between days P2-P10 resulted in a phenotype similar to that observed with Hnf6 knockout, but with slower kinetics [158]. Notably, germline inactivation of LKB1 is the cause of Peutz-Jeghers syndrome, which is associated with a highly elevated risk of pancreatic cancer [33], and Lkb1 has also been associated with ciliary signaling [158].

*ARF4.* The small GTPase ARF4 was originally proposed to function in regulating trafficking of proteins to the cilium, leading Pearring and colleagues to investigate its activity relevant to common ciliopathies [165]. Although extensive analysis using induce organ-specific and complete loss of ARF4 suggested that ARF4 does not have an essential role at cilia, although it is essential for trafficking of proteins between the endoplasmic reticulum and Golgi, essential for polarized secretion of digestive enzymes. Strikingly, in a whole-body knockout model with loss of ARF4 induced at postnatal day P2, the most dramatic phenotype observed was profound loss of acinar cells, with extensive replacement by adipocytes and fibrosis already notable by day P10 [165]. While no cells co-expressing lipid droplets and zymogen granules were observed (as might be expected in intermediate states, if transdifferentiation occurred between acinar cells and adipocytes), the authors noted that zymogen granules were lost very rapidly, so it was not possible to make a determination of the source of the adipocytes. Endocrine cells and organization were unaffected.

*IKKα and NF-κB.* The kinase IKKα phosphorylates IKK proteins, which are negative regulators of NF-κB, a transcription factor that induces transcription of cytokines and survival factors. Early studies of NF-κB showed that too much NF-κB in acinar cells causes pancreatitis [166] whereas too little NF-κB activity sensitizes acinar cells to cerulein treatment [167], emphasizing the need to maintain NF-κB activity in a narrow range for pancreatic homeostasis. In a detailed analysis of mice in which Pdx1-Cre was used to ablate IKKα function, mice developed extensive acinar cell vacuolization, inter- and intralobular fibrosis, ductal metaplasia, immune cell infiltration, and acinar atrophy with adipose replacement of acinar cells, but also increased proliferation in areas of surviving acinar cells, and signs of ADM [168]. Notably, these activities did not reflect loss of kinase activity and control of NF-κB, but rather kinase independent role of IKKα in regulating autophagic degradation; simultaneous ablation of p62 in mice with pancreatic knockout of IKKα showed reduced signs of oxidative and ER stress, and had reduced symptoms of pancreatitis; parallel examination of human pancreatitis specimens suggested many were characterized by downregulation of IKKα and elevated p62, implying clinical relevance of the findings [168].

*UBIAD1*. Vitamin K (representing phylloquinone and a group of menaquinones) is a cofactor for enzymes involved in diverse cellular processes. UbiA prenyltransferase domain-containing protein 1 (UBIAD1) is an enzyme involved in vitamin K synthesis, and additionally regulates the synthesis of cholesterol. Induced knockout of Ubiad1 in young mice led to lethality within two months; however, this was preceded by rapid pancreatic reprogramming characterized by acinar cell apoptosis and loss, and replacement by adipocytes. This was accompanied by striking upregulation of PPARγ and markers of oxidative stress. By contrast, islets were largely unaffected [169].

*TGFβ*. Signaling by transforming growth factor beta (TGFβ) has long attracted interest for developmental and cancer biologists given early recognition that this ligand can be either growth-inhibitory or growth-promoting based on cellular context. A 1997 study [170] assessed the effect of dominant negative overexpression of TGFBRII (a primary receptor for TGFβ) under the control of an inducible MT1 promoter active in the whole body. This caused multiple consequences with the pancreas most strongly affected among organs; observed pancreatic phenotypes included accumulation of ducts (indicative of acinar-ductal metaplasia, ADM), fibrosis, and replacement of acinar cells with adipocytes. Notably, surviving acinar cells expressed elevated levels of TGFβ1 and TGFβ3, likely reflecting reaction to cell-intrinsic reduced activity of the pathway, and increasing levels of pathway-activating ligands in the vicinity of acinar cells. This study would lead to the straightforward conclusion that TGFβ signaling is necessary for acinar cell viability and to suppress pathological adipocyte infiltration.

However, this interpretation is complicated by a more recent study in which a constitutively active form of TGFBRI (the heterodimerizing partner of TGFBRII) was similarly overexpressed again, with the pancreas again most strongly affected [171]. Detailed analysis of overexpression of TGFBRI^CA^ in the pancreas using a *Pdx1* promoter, or selectively in acinar cells from adult mice using a *Ptf1a-Cre*^*ERT*^ system, resulted in acinar loss, evidence of ADM, and accumulation of adipocytes. This would support the opposite conclusion; that too much TGFβ signaling is deleterious to acinar viability, and promotes ADM. The conflicting results remain to be resolved; however, one striking result of the latter study was the contrast between biological outcomes in expression of TGFBRI^CA^ in acinar cells alone, versus in acinar cells expressing an activating allele of *KRAS*. In the latter case, no formation of adipocytes was observed; rather, enhanced the formation of PanINs induced by oncogenic KRAS [171]. This provides strong evidence for TGFβ-dependent adipogenesis being directly regulated within acinar cells, dependent on the presence or absence of a transformational stimulus. Parallel experiments in vitro indicated TGFβ and RAS collaborated to induce ADM in purified acinar cells; unfortunately, whether TGFBRI^CA^ and KRAS interacted to influence TGFβ production in the in vivo setting was not assessed.

### IPAs and mouse models of PDAC

Mouse models are a mainstay of PDAC research, with multiple models for induction of tumorigenesis in current use to support analysis of the tumorigenic process [172]. Most of these models use pancreatic-specific promoters to induce activation of KRAS or other oncogenes, alone or in combination, and studies focus on assessment of tumors. Typically, in mouse models, tumors arise 3–6 months after introduction of an oncogene, and have many features of human PDACs, indicating at least partial simulation of the human pathogenic processes. As with human PDACs, IPAs are not abundant within the mass of the established tumor, and IPA accumulation or action early in the tumorigenic process are not usually a focus of study (or at least, are typically not reported). The unexpected diversity of factors causing acinar loss and IPA accumulation summarized above, which include many factors of relevance to PDAC studies, suggest studies of mechanisms of tumorigenesis may benefit from such analysis. These results also suggest ways in which induction method used for manipulation of genes that induce oncogenesis may indirectly affect outcomes for tumor formation.

Of the examples of IPA accumulation cited above for mice, some but not all of these genetic mouse models can be characterized into groups based on overlapping affected pathways (Fig. 7), and there is still a lack of experimental data indicating what changes in molecular signaling cause exocrine atrophy, altered pancreatic differentiation, and accumulation of adipocytes. Importantly, genetic models often use promoters that are constitutively active throughout pancreatic formation (*Pdx*), or during development in acinar cells (*Ptf, Ela1*) or ductal cells (*Hnf6*) during late development; in some cases, incorporation of an inducible Cre under control of these promoters allows more exact timing of genetic manipulation in adult animals. Some induce severe defects in embryonal morphogenesis, and are accompanied by pancreatic defects before or proximal to birth. Leaving these aside, a common profile is a pancreas that is typically normal at birth, but where IPAs begin to be apparent in the tissue by several weeks of age, and dominate in the organ after a few months. Genetic changes that give rise to IPA in young adult animals include genes that promote proliferation and de-differentiation (MYC); that influence acinar cell maturation (Notch); that promote ER stress and trafficking (UBIAD, IKKα and probably ARF4 [173]); that influence the inflammatory microenvironment (TGFβ and periostin); and that interfere with ductal formation and function, often through targeting of cilia, which serve both mechanosensing and signal transduction (NOTCH, KIF3A, IFT88, CHBY, and associated signaling proteins). Provocatively, all these processes would be engaged in response to acute pancreatitis, in which ductal blockade interfered with acinar cell homeostasis.

In this context, it is also intriguing that activating KRAS mutations are nearly ubiquitous in human PDAC, and that activated KRAS has distinct transforming properties when expressed in ductal cells, which are ciliated, and acinar cells, which are not [115]. KRAS activation causes loss of ciliation by activating AURKA, a proximal inducer of ciliary loss [174, 175]. In cancer cells, KRAS-induced loss of cilia establishes asymmetry versus ciliated mesenchymal cells in the tumor microenvironment, affecting ability to respond to Hh, which selectively activates CAFs to produce the extracellular matrix and soluble factors required to support tumor growth [176]. In the normal pancreas, KRAS activation in ductal cells would cause loss of ductal mechanosensory signaling and response to factors such as Hh, a key morphogen and regulator of tissue homeostasis and regeneration [177]. Notably, the ADM following acinar cell injury is marked by expression of cilia on emerging ductal cells, which allows cells to respond to HH to regenerate; this process is blocked by KRAS expression, but restored by AURKA inhibition [178]. Given that in most mouse models used to study PDAC, KRAS is expressed under the control of broadly acting promoters, in concept KRAS disruption of cilia would occur simultaneously in enough cells to have a biologically significant disruptive effect on ductal function, simulating the acinar insult induced by acute pancreatitis, and potentially contributing to IPA accumulation. Whether such an activity exists or is relevant to physiological human PDAC, where activating KRAS mutations would occur in isolated cells over time, remains to be established. Certainly, control of ciliation would not be the only way in which KRAS could affect IPA and lipid droplet abundance; for example, one recent study has shown that expression of KRAS in acinar cells reduces expression of FGF21, a metabolic regulator that reduces obesity, and that treatment of KRAS^G12D/+^ mice maintained on a high fat diet with recombinant FGF21 reduced pancreatic triglyceride levels, inflammation, and tumorigenesis [44]. The activity of KRAS in the context of cancer risk factors in pre-malignant tissue would benefit from considerable further investigation.

## Humans versus mice: unanswered questions

In spite of the many invaluable lessons gained from use of mouse models, there are obvious ways in which pathogenesis of human PDAC differs, driven foremost by the difference in murine and human lifespans. Most murine PDAC is studied in young healthy mice, and occurs in months. In humans, premalignant lesions and convert to PDAC over decades, rather than months, in the context of aging and often, increasing obesity, which result in a gradual accumulation of IPAs and lipid droplets, as well as many other changes. The predisposing condition of acute pancreatitis induced in mice causes rapid, broadscale death of acinar cells and can cause similarly rapid replacement with IPAs when induced by ductal blockage, but not when induced by cerulein. In contrast, acute pancreatitis can arise from more focal events, and sequelae affecting tissue composition are less well studied. Although numerous studies report linkages between pancreatitis, IPAs, fibrosis, and early cancerous lesions, causality is not yet understood. Whether PanINs associate with areas of parenchymal damage because signals from the damaged tissue promote PanIN formation, or whether the PanINs form and produce signals that lead to tissue damage, including IPA replacement of acinar cells, remains unclear.

Many studies over the past decades have used mouse models maintained on high fat diets or with genetic lesions promoting obesity to study PDAC origins, typically finding a significant tumor-promoting effect that parallels associations seen in human [12, 13, 179]. In these models, changing levels of IPAs are one of a suite of induced changes, including altered systemic effects, and exocrine-endocrine signaling, that interact with genetic activation of PDAC typically induced simultaneously in many cancer precursor cells, rather than the rare clonal initiating events seen in humans. Whether the timing of events and stoichiometry of interacting cell populations in these models simulates the mechanisms seen in humans remains to be determined. A further critical difference between most mouse models and humans is the overall condition of the pancreatic milieu associated with aging. Studies of cancer formation in aged mice are currently limited; nevertheless, a growing body of work demonstrates very significant changes in the composition of tumor microenvironments, including handling of lipids, in ways that can strikingly affect tumor progression, metastasis, and therapeutic resistance [180–182]. Studies of PDAC initiation in older mice, using promoters designed to achieve sporadic rather than global activation of *KRAS*, are likely to come closer to approximating the events occurring in the aging human pancreas [21, 22, 57].

Importantly, the diversity of genetic defects leading to IPA accumulation in mice is extremely surprising, and the implications of these findings for human pancreatitis and PDAC remain in large part obscure, and merit further study. Given ambiguity in reports as to the source of IPAs (transdifferentiation or infiltration), a systematic investigation of whether there is functional diversity among IPAs observed in the pancreas, and whether this changes during tumor formation, would be valuable. Of particular interest is implication of defects in numerous genes involved in regulating ciliary function in accumulation of IPAs. There are a large number of ciliopathies in humans, arising from inherited mutations in genes affecting cilia formation and signaling; symptoms associated with ciliopathies include obesity and pancreatic dysfunction [183]. Given the relative rarity of ciliopathies in the general population, and the fact that many ciliopathies cause severe symptoms that manifest early in life, the potential role of cilia in influencing the pancreatic milieu has not been previously studied. However, one intriguing connection may be through considering the role of Hedgehog (Hh) signaling, which proceeds through a ciliary receptor, in pancreatitis and pancreatic cancer. Loss of Hh prevents regeneration of acinar in response to cerulein and promotes acinar metaplasia similar to that in ADM [184]. Interestingly, Hh signaling also reduces the degree of adiposity induced by high fat diets in rodents [185], in a non-cancer setting. In contrast, Hh-dependent signaling in fibroblasts and immune cells plays a complex role in pancreatic desmoplasia, promoting while constraining the growth of PDACs [186, 187]. It would be of considerable interest to investigate how this signaling axis may influence IPAs in the pre-malignant human pancreas.

## Summary

The past decade has been characterized by the rising recognition of the importance of cancer cell interactions with constituents of the tumor microenvironment [188], as well as the importance of metabolic reprogramming [189], as factors specifying tumor aggressiveness and therapeutic response. While these topics are now the subject of extensive study for pancreatic and other cancers, much of this research focuses on changes in cellularity accompanying or subsequent to the emergence of an established tumor mass. In parallel, there has been rising recognition that risk for many cancers arises from modifiable factors, including obesity, and that cancer incidence and mortality can be sharply reduced by understanding and addressing the action of these factors, which condition the environment in which tumors arise [190]. Because these risk factors act before tumors arise, how they influence the early stages of tumorigenesis are more elusive. This is particularly a concern for PDAC, which is typically identified at a very advanced stage in humans. Given the complex and changing cellularity of the pancreas pre- and post-tumor formation, the origin and role of IPAs in PDAC tumor risk and tumor pathogenesis remains poorly defined.

As discussed here, many studies in mouse models suggest that IPAs are induced by multiple forms of pancreatic dysfunction that cause changes in pancreatic acinar and ductal cell composition, with evidence suggesting more than one type of cell can serve as an IPA precursor. Whether similar mechanisms leading to IPAs occur in humans is largely unknown. Both patient data and studies in mouse models show IPA accumulation to be associated with both acute and chronic pancreatitis. While these data clearly link increased IPAs to the risk of developing pancreatitis, how this adipocyte population contributes to the pro-inflammatory environment and exocrine atrophy observed in mouse models is poorly understood, and there is a complete dearth of studies investigating IPA accumulation in the setting of cerulein-induced pancreatitis, a commonly employed mouse model of pancreatitis. Given the evidence of how ectopic fat deposits affect other tissues, such as the liver, IPAs may promote the storage and secretion of factors that affect metabolism and inflammation of pancreatic cells to promote initiation and progression of pancreatitis. Similarly, the increase in IPAs appears likely to increase pancreatic tumor risk in part through its role in promoting pancreatitis, as over time, these changes may promote an environment in which pre-malignant cancer cells can thrive and progress. Because of this, a better understanding of the origin and activity of IPAs is critical to correctly interpret the ultimate cancer phenotypes obtained with PDAC mouse models, and to understand how IPAs may promote PDAC in patients. Importantly, increased scrutiny of the interaction between IPAs, other risk factors, and oncogene activity in the premalignant setting has the potential to yield insights valuable for pancreatic cancer prevention and cancer interception, and merits further investigation.

## Data Availability

Not applicable.

## References

[1] World Health Organization. *Obesity and overweight*. 9 June 2021; https://www.who.int/en/news-room/fact-sheets/detail/obesity-and-overweight.

[2] Wu H, Ballantyne CM (2020). Metabolic inflammation and insulin resistance in obesity. Circ Res.

[3] Fryar CD, Carroll MD, Afful J Prevalence of Overweight, Obesity, and Severe Obesity Among Adults Aged 20 and Over: United States, 1960–1962 Through 2017–2018. 2021, National Center for Health Statisitics. 41779887

[4] Avgerinos KI (2019). Obesity and cancer risk: Emerging biological mechanisms and perspectives. Metabolism.

[5] Zatterale F (2019). Chronic adipose tissue inflammation linking obesity to insulin resistance and type 2 diabetes. Front Physiol.

[6] Iannuzzi JP (2022). Global Incidence of acute pancreatitis is increasing over time: a systematic review and meta-analysis. Gastroenterology.

[7] Beyer G (2020). Chronic pancreatitis. Lancet.

[8] Klein AP (2021). Pancreatic cancer epidemiology: understanding the role of lifestyle and inherited risk factors. Nat Rev Gastroenterol Hepatol.

[9] Navina S et al. (2011) Lipotoxicity causes multisystem organ failure and exacerbates acute pancreatitis in obesity*.* Sci Transl Med. 3(107): 107ra110 10.1126/scitranslmed.3002573 10.1126/scitranslmed.3002573 PMC3321362 22049070

[10] van den Berg FF (2021). Western-type diet influences mortality from necrotising pancreatitis and demonstrates a central role for butyrate. Gut.

[11] Incio J (2016). Obesity-induced inflammation and desmoplasia promote pancreatic cancer progression and resistance to chemotherapy. Cancer Discov.

[12] Chung KM et al. (2020) Endocrine-Exocrine Signaling Drives Obesity-Associated Pancreatic Ductal Adenocarcinoma. Cell. 181(4): 832–847 e18 10.1016/j.cell.2020.03.062 10.1016/j.cell.2020.03.062 PMC7266008 32304665

[13] Khasawneh J et al. (2009) Inflammation and mitochondrial fatty acid beta-oxidation link obesity to early tumor promotion. Proc Natl Acad Sci USA. 106(9): 3354–9 10.1073/pnas.0802864106 10.1073/pnas.0802864106 PMC2651311 19208810

[14] da Cruz RS (2019). Parental obesity programs pancreatic cancer development in offspring. Endocr Relat Cancer.

[15] Storz P (2017). Acinar cell plasticity and development of pancreatic ductal adenocarcinoma. Nat Rev Gastroenterol Hepatol.

[16] Kirkegard J, Mortensen FV, Cronin-Fenton D (2017). Chronic pancreatitis and pancreatic cancer risk: a systematic review and meta-analysis. Am J Gastroenterol.

[17] Ye J (2013). Mechanisms of insulin resistance in obesity. Front Med.

[18] Mauvais-Jarvis F (2016). Role of sex steroids in beta cell function, growth, and survival. Trends Endocrinol Metab.

[19] Petrov MS, Taylor R (2022). Intra-pancreatic fat deposition: bringing hidden fat to the fore. Nat Rev Gastroenterol Hepatol.

[20] Shulman GI (2014). Ectopic fat in insulin resistance, dyslipidemia, and cardiometabolic disease. N Engl J Med.

[21] Matsuda Y (2018). Age-related pathological changes in the pancreas. Front Biosci (Elite Ed).

[22] Lohr JM (2018). The ageing pancreas: a systematic review of the evidence and analysis of the consequences. J Intern Med.

[23] Tardif N (2014). Muscle ectopic fat deposition contributes to anabolic resistance in obese sarcopenic old rats through eIF2alpha activation. Aging Cell.

[24] Lankisch PG, Apte M, Banks PA (2015). Acute pancreatitis. Lancet.

[25] Lee PJ, Papachristou GI (2019). New insights into acute pancreatitis. Nat Rev Gastroenterol Hepatol.

[26] de Oliveira C (2020). Pancreatic triglyceride lipase mediates lipotoxic systemic inflammation. J Clin Invest.

[27] Khatua B, El-Kurdi B, Singh VP (2017). Obesity and pancreatitis. Curr Opin Gastroenterol.

[28] Durgampudi C (2014). Acute lipotoxicity regulates severity of biliary acute pancreatitis without affecting its initiation. Am J Pathol.

[29] Natu A (2017). Visceral adiposity predicts severity of acute pancreatitis. Pancreas.

[30] Kleeff J (2017). Chronic pancreatitis. Nat Rev Dis Primers.

[31] Kleeff J (2016). Pancreatic cancer. Nat Rev Dis Primers.

[32] van Lier MG et al. (2010) High cancer risk in Peutz-Jeghers syndrome: a systematic review and surveillance recommendations. Am J Gastroenterol. 105(6): 1258–64; author reply 1265 10.1038/ajg.2009.725 10.1038/ajg.2009.725 20051941

[33] Giardiello FM (2000). Very high risk of cancer in familial Peutz-Jeghers syndrome. Gastroenterology.

[34] Resta N (2013). Cancer risk associated with STK11/LKB1 germline mutations in Peutz-Jeghers syndrome patients: results of an Italian multicenter study. Dig Liver Dis.

[35] Shackelford DB, Shaw RJ (2009). The LKB1-AMPK pathway: metabolism and growth control in tumour suppression. Nat Rev Cancer.

[36] Alexander J, Cukierman E (2020). Cancer associated fibroblast: mediators of tumorigenesis. Matrix Biol.

[37] Perera RM (2015). Transcriptional control of autophagy-lysosome function drives pancreatic cancer metabolism. Nature.

[38] Commisso C (2013). Macropinocytosis of protein is an amino acid supply route in Ras-transformed cells. Nature.

[39] Iacobuzio-Donahue CA (2012). Genetic evolution of pancreatic cancer: lessons learnt from the pancreatic cancer genome sequencing project. Gut.

[40] Makohon-Moore AP (2017). Limited heterogeneity of known driver gene mutations among the metastases of individual patients with pancreatic cancer. Nat Genet.

[41] Means AL (2005). Pancreatic epithelial plasticity mediated by acinar cell transdifferentiation and generation of nestin-positive intermediates. Development.

[42] Kopp JL (2012). Identification of Sox9-dependent acinar-to-ductal reprogramming as the principal mechanism for initiation of pancreatic ductal adenocarcinoma. Cancer Cell.

[43] Flowers BM (2021). Cell of origin influences pancreatic cancer subtype. Cancer Discov.

[44] Luo Y, et al. (2019) Oncogenic KRAS Reduces Expression of FGF21 in Acinar Cells to Promote Pancreatic Tumorigenesis in Mice on a High-Fat Diet*.* Gastroenterology. 157(5): 1413–1428 e11 10.1053/j.gastro.2019.07.030 10.1053/j.gastro.2019.07.030 PMC6815712 31352001

[45] Gidekel Friedlander SY (2009). Context-dependent transformation of adult pancreatic cells by oncogenic K-Ras. Cancer Cell.

[46] Bailey JM (2016). p53 mutations cooperate with oncogenic Kras to promote adenocarcinoma from pancreatic ductal cells. Oncogene.

[47] Boj SF (2015). Organoid models of human and mouse ductal pancreatic cancer. Cell.

[48] Moffitt RA (2015). Virtual microdissection identifies distinct tumor- and stroma-specific subtypes of pancreatic ductal adenocarcinoma. Nat Genet.

[49] Wollny D (2016). Single-cell analysis uncovers clonal acinar cell heterogeneity in the adult pancreas. Dev Cell.

[50] Hendley AM et al. (2021) Single-cell transcriptome analysis defines heterogeneity of the murine pancreatic ductal tree*.* Elife. 10 10.7554/eLife.67776 10.7554/eLife.67776 PMC8184217 34009124

[51] Guerra C (2007). Chronic pancreatitis is essential for induction of pancreatic ductal adenocarcinoma by K-Ras oncogenes in adult mice. Cancer Cell.

[52] Habbe N (2008). Spontaneous induction of murine pancreatic intraepithelial neoplasia (mPanIN) by acinar cell targeting of oncogenic Kras in adult mice. Proc Natl Acad Sci U S A.

[53] Ma Z, et al. (2022) Single-cell transcriptomics reveals a conserved metaplasia program in pancreatic injury. Gastroenterology. 162(2): 604–620 e20 10.1053/j.gastro.2021.10.027 10.1053/j.gastro.2021.10.027 PMC8792222 34695382

[54] Tosti L et al. (2021) Single-Nucleus and In Situ RNA-Sequencing Reveal Cell Topographies in the Human Pancreas*.* Gastroenterology. 160(4): 1330–1344 e11 10.1053/j.gastro.2020.11.010 10.1053/j.gastro.2020.11.010 33212097

[55] Cobo I (2018). Transcriptional regulation by NR5A2 links differentiation and inflammation in the pancreas. Nature.

[56] Hoang CQ et al. (2016) Transcriptional maintenance of pancreatic acinar identity, Differentiation, and Homeostasis by PTF1A. Mol Cell Biol. 36(24): 3033–3047 10.1128/MCB.00358-16 10.1128/MCB.00358-16 PMC5126291 27697859

[57] Moin ASM et al. (2020) Pancreatic alpha-cell mass across adult human lifespan. Eur J Endocrinol. 182(2): 219–231 10.1530/EJE-19-0844 10.1530/EJE-19-0844 PMC6944979 31821160

[58] Brune K (2006). Multifocal neoplastic precursor lesions associated with lobular atrophy of the pancreas in patients having a strong family history of pancreatic cancer. Am J Surg Pathol.

[59] Rebours V et al. (2015) Obesity and fatty pancreatic infiltration are risk factors for pancreatic precancerous lesions (PanIN)*.* Clin Cancer Res. 21(15): 3522–8 10.1158/1078-0432.CCR-14-2385 10.1158/1078-0432.CCR-14-2385 25700304

[60] Olsen, T.S. (1978) Lipomatosis of the pancreas in autopsy material and its relation to age and overweight. Acta Pathol Microbiol Scand A. 86A(5): 367–73 10.1111/j.1699-0463.1978.tb02058.x 10.1111/j.1699-0463.1978.tb02058.x 716899

[61] Smits MM, van Geenen EJ (2011). The clinical significance of pancreatic steatosis. Nat Rev Gastroenterol Hepatol.

[62] Truong E, Pandol S, Jeon C (2022) Uniting epidemiology and experimental models: pancreatic steatosis and pancreatic cancer. EBioMedicine. 79: 103996 10.1016/j.ebiom.2022.103996 10.1016/j.ebiom.2022.103996 PMC9010750 35405390

[63] Stamm BH (1984). Incidence and diagnostic significance of minor pathologic changes in the adult pancreas at autopsy: a systematic study of 112 autopsies in patients without known pancreatic disease. Hum Pathol.

[64] Kromrey ML (2019). Pancreatic steatosis is associated with impaired exocrine pancreatic function. Invest Radiol.

[65] Kuhn JP (2015). Pancreatic steatosis demonstrated at MR imaging in the general population: clinical relevance. Radiology.

[66] Wang CY (2014). Enigmatic ectopic fat: prevalence of nonalcoholic fatty pancreas disease and its associated factors in a Chinese population. J Am Heart Assoc.

[67] Staaf J (2017). Pancreatic fat is associated with metabolic syndrome and visceral fat but not beta-cell function or body mass index in pediatric obesity. Pancreas.

[68] Lee JS (2009). Clinical implications of fatty pancreas: correlations between fatty pancreas and metabolic syndrome. World J Gastroenterol.

[69] Smith U (2015). Abdominal obesity: a marker of ectopic fat accumulation. J Clin Invest.

[70] Wagner R (2022). Metabolic implications of pancreatic fat accumulation. Nat Rev Endocrinol.

[71] Stuart CE (2020). Implications of tobacco smoking and alcohol consumption on ectopic fat deposition in individuals after pancreatitis. Pancreas.

[72] Ko J (2022). Associations between intra-pancreatic fat deposition, pancreas size, and pancreatic enzymes in health and after an attack of acute pancreatitis. Obes Facts.

[73] Fujii M (2019). Impact of fatty pancreas and lifestyle on the development of subclinical chronic pancreatitis in healthy people undergoing a medical checkup. Environ Health Prev Med.

[74] Tirkes T (2019). Association of pancreatic steatosis with chronic pancreatitis, obesity, and type 2 diabetes mellitus. Pancreas.

[75] Tirkes T (2022). Quantitative MRI of chronic pancreatitis: results from a multi-institutional prospective study, magnetic resonance imaging as a non-invasive method for assessment of pancreatic fibrosis (MINIMAP). Abdom Radiol (NY)..

[76] Sreedhar UL (2020). A systematic review of intra-pancreatic fat deposition and pancreatic carcinogenesis. J Gastrointest Surg.

[77] Kanno A (2018). Multicenter study of early pancreatic cancer in Japan. Pancreatology.

[78] Khoury, T. and W. Sbeit. (2022) *Fatty Pancreas and Pancreatic Cancer: An Overlooked Association?* J Clin Med. **11**(3) 10.3390/jcm11030763 10.3390/jcm11030763 PMC8836883 35160214

[79] Venet T (2017). Severe infantile isolated exocrine pancreatic insufficiency caused by the complete functional loss of the SPINK1 gene. Hum Mutat.

[80] Singhi AD (2014). The histopathology of PRSS1 hereditary pancreatitis. Am J Surg Pathol.

[81] Mishra A, et al. (2021) Perilipin 2 downregulation in beta cells impairs insulin secretion under nutritional stress and damages mitochondria. JCI Insight. 6(9) 10.1172/jci.insight.144341 10.1172/jci.insight.144341 PMC8262280 33784258

[82] Pinnick KE (2008). Pancreatic ectopic fat is characterized by adipocyte infiltration and altered lipid composition. Obesity (Silver Spring).

[83] Parry EW (1972). Lipid in pancreatic exocrine cells of rats bearing the Walker tumour. Br J Cancer.

[84] Yan MX (2006). Long-term high-fat diet induces pancreatic injuries via pancreatic microcirculatory disturbances and oxidative stress in rats with hyperlipidemia. Biochem Biophys Res Commun.

[85] Pothula SP (2020). Pancreatic stellate cells: Aiding and abetting pancreatic cancer progression. Pancreatology.

[86] Ozdemir BC (2014). Depletion of carcinoma-associated fibroblasts and fibrosis induces immunosuppression and accelerates pancreas cancer with reduced survival. Cancer Cell.

[87] Sunami Y, Rebelo A, Kleeff J (2017) Lipid Metabolism and Lipid Droplets in Pancreatic Cancer and Stellate Cells*.* Cancers (Basel). 10(1) 10.3390/cancers10010003 10.3390/cancers10010003 PMC5789353 29295482

[88] Olzmann JA, Carvalho P (2019). Dynamics and functions of lipid droplets. Nat Rev Mol Cell Biol.

[89] Ackerman D et al. (2018) Triglycerides promote lipid homeostasis during hypoxic stress by balancing fatty acid saturation. Cell Rep. 24(10): 2596–2605 e5 10.1016/j.celrep.2018.08.015 10.1016/j.celrep.2018.08.015 PMC6137821 30184495

[90] Noel P (2016). Peripancreatic fat necrosis worsens acute pancreatitis independent of pancreatic necrosis via unsaturated fatty acids increased in human pancreatic necrosis collections. Gut.

[91] Garcia-Rayado G (2020). Dietary fat patterns and outcomes in acute pancreatitis in Spain. Front Med (Lausanne)..

[92] Chang YT (2015). Distinctive roles of unsaturated and saturated fatty acids in hyperlipidemic pancreatitis. World J Gastroenterol.

[93] Biczo G (2018). Mitochondrial dysfunction, through impaired autophagy, leads to endoplasmic reticulum stress, deregulated lipid metabolism, and pancreatitis in animal models. Gastroenterology.

[94] Ravaut G et al. (2020) Monounsaturated Fatty Acids in Obesity-Related Inflammation*.* Int J Mol Sci. 22(1) 10.3390/ijms22010330 10.3390/ijms22010330 PMC7795523 33396940

[95] Sbeit W, Khoury T (2021). Fatty pancreas represents a risk factor for acute pancreatitis: a pilot study. Pancreas.

[96] Renehan AG, Zwahlen M, Egger M (2015). Adiposity and cancer risk: new mechanistic insights from epidemiology. Nat Rev Cancer.

[97] Hingorani, S.R. (2022) *Epithelial and stromal co-evolution and complicity in pancreatic cancer.* Nat Rev Cancer. 10.1038/s41568-022-00530-w 10.1038/s41568-022-00530-w PMC10470605 36446904

[98] Rozeveld CN (2020). KRAS controls pancreatic cancer cell lipid metabolism and invasive potential through the lipase HSL. Cancer Res.

[99] Auciello FR (2019). A stromal lysolipid-autotaxin signaling axis promotes pancreatic tumor progression. Cancer Discov.

[100] Huang BZ et al. (2017) Influence of Statins and Cholesterol on Mortality Among Patients With Pancreatic Cancer. J Natl Cancer Inst. 109(5) 10.1093/jnci/djw275 10.1093/jnci/djw275 28040693

[101] Gabitova-Cornell L et al. (2020) Cholesterol Pathway Inhibition Induces TGF-β Signaling to Promote Basal Differentiation in Pancreatic Cancer*.* Cancer Cell. 10.1016/j.ccell.2020.08.015 10.1016/j.ccell.2020.08.015 PMC7572882 32976774

[102] Karasinska JM (2020). Altered gene expression along the glycolysis-cholesterol synthesis axis is associated with outcome in pancreatic cancer. Clin Cancer Res.

[103] Gouw AM et al. (2019) The MYC oncogene cooperates with sterol-regulated element-binding protein to regulate lipogenesis essential for neoplastic growth*.* Cell Metab. 30(3): 556–572 e5 10.1016/j.cmet.2019.07.012 10.1016/j.cmet.2019.07.012 PMC6911354 31447321

[104] Ko J et al. (2022) Intrapancreatic, liver, and skeletal muscle fat depositions in first attack of acute pancreatitis versus health. Am J Gastroenterol 117(10): 1693–1701 10.14309/ajg.0000000000001951 10.14309/ajg.0000000000001951 35971231

[105] Majumder S (2017). Fatty pancreas: Should we be concerned?. Pancreas.

[106] Catanzaro R (2016). Exploring the metabolic syndrome: nonalcoholic fatty pancreas disease. World J Gastroenterol.

[107] Fraulob JC (2010). A mouse model of metabolic syndrome: insulin resistance, fatty liver and non-alcoholic fatty pancreas disease (NAFPD) in C57BL/6 mice fed a high fat diet. J Clin Biochem Nutr.

[108] Gotoh K (2012). Spleen-derived interleukin-10 downregulates the severity of high-fat diet-induced non-alcoholic fatty pancreas disease. PLoS ONE.

[109] Mathur A (2007). Nonalcoholic fatty pancreas disease. HPB (Oxford).

[110] Peng C (2022). Murine chronic pancreatitis model induced by partial ligation of the pancreatic duct encapsulates the profile of macrophage in human chronic pancreatitis. Front Immunol.

[111] Watanabe S (1995). Changes in the mouse exocrine pancreas after pancreatic duct ligation: a qualitative and quantitative histological study. Arch Histol Cytol.

[112] Geisz A, Sahin-Toth M (2018). A preclinical model of chronic pancreatitis driven by trypsinogen autoactivation. Nat Commun.

[113] Hausmann S (2016). Loss of periostin results in impaired regeneration and pancreatic atrophy after cerulein-induced pancreatitis. Am J Pathol.

[114] Cano DA (2014). Transcriptional control of mammalian pancreas organogenesis. Cell Mol Life Sci.

[115] diIorio P (2014). Role of cilia in normal pancreas function and in diseased states. Birth Defects Res C Embryo Today.

[116] Yu XX et al. (2019) Defining multistep cell fate decision pathways during pancreatic development at single-cell resolution. EMBO J. 38(8) 10.15252/embj.2018100164 10.15252/embj.2018100164 PMC6463266 30737258

[117] Larsen HL, Grapin-Botton A (2017). The molecular and morphogenetic basis of pancreas organogenesis. Semin Cell Dev Biol.

[118] Lodestijn SC et al. (2021) Continuous clonal labeling reveals uniform progenitor potential in the adult exocrine pancreas. Cell Stem Cell. 28(11): 2009–2019 e4 10.1016/j.stem.2021.07.004 10.1016/j.stem.2021.07.004 PMC8577826 34358441

[119] Grimont A, Leach SD, Chandwani R (2022). Uncertain beginnings: acinar and ductal cell plasticity in the development of pancreatic cancer. Cell Mol Gastroenterol Hepatol.

[120] Westmoreland JJ et al. (2012) Pancreas-specific deletion of Prox1 affects development and disrupts homeostasis of the exocrine pancreas*.* Gastroenterology. 142(4): 999–1009 e6 10.1053/j.gastro.2011.12.007 10.1053/j.gastro.2011.12.007 PMC3398795 22178591

[121] Wang J (2005). Prox1 activity controls pancreas morphogenesis and participates in the production of "secondary transition" pancreatic endocrine cells. Dev Biol.

[122] Jonsson J (1994). Insulin-promoter-factor 1 is required for pancreas development in mice. Nature.

[123] Stoffers DA (1997). Pancreatic agenesis attributable to a single nucleotide deletion in the human IPF1 gene coding sequence. Nat Genet.

[124] Heller RS (2001). Improved glucose tolerance and acinar dysmorphogenesis by targeted expression of transcription factor PDX-1 to the exocrine pancreas. Diabetes.

[125] Xuan S (2012). Pancreas-specific deletion of mouse Gata4 and Gata6 causes pancreatic agenesis. J Clin Invest.

[126] Carrasco M (2012). GATA4 and GATA6 control mouse pancreas organogenesis. J Clin Invest.

[127] Decker K (2006). Gata6 is an important regulator of mouse pancreas development. Dev Biol.

[128] Martinelli P (2013). Gata6 is required for complete acinar differentiation and maintenance of the exocrine pancreas in adult mice. Gut.

[129] Dessimoz J (2005). Pancreas-specific deletion of beta-catenin reveals Wnt-dependent and Wnt-independent functions during development. Curr Biol.

[130] Murtaugh LC (2005). Beta-catenin is essential for pancreatic acinar but not islet development. Development.

[131] Wells JM (2007). Wnt/beta-catenin signaling is required for development of the exocrine pancreas. BMC Dev Biol.

[132] Strom A (2007). Unique mechanisms of growth regulation and tumor suppression upon Apc inactivation in the pancreas. Development.

[133] Bonal C et al. (2009) Pancreatic inactivation of c-Myc decreases acinar mass and transdifferentiates acinar cells into adipocytes in mice. Gastroenterology. 136(1): 309–319 e9 10.1053/j.gastro.2008.10.015 10.1053/j.gastro.2008.10.015 19022256

[134] Nakhai H (2008). Conditional inactivation of Myc impairs development of the exocrine pancreas. Development.

[135] Sánchez-Arévalo Lobo VJ (2018). c-Myc downregulation is required for preacinar to acinar maturation and pancreatic homeostasis. Gut.

[136] Masui T (2010). Replacement of Rbpj with Rbpjl in the PTF1 complex controls the final maturation of pancreatic acinar cells. Gastroenterology.

[137] Wallace K (2010). Disrupted pancreatic exocrine differentiation and malabsorption in response to chronic elevated systemic glucocorticoid. Am J Pathol.

[138] Carver EA (2001). The mouse snail gene encodes a key regulator of the epithelial-mesenchymal transition. Mol Cell Biol.

[139] Loubat-Casanovas J et al. (2016) Snail1 is required for the maintenance of the pancreatic acinar phenotype. Oncotarget. 7(4): 4468–82 10.18632/oncotarget.6785 10.18632/oncotarget.6785 PMC4826219 26735179

[140] Grippo PJ, Sandgren EP (2012). Acinar-to-ductal metaplasia accompanies c-myc-induced exocrine pancreatic cancer progression in transgenic rodents. Int J Cancer.

[141] Gonzalez-Gonzalez, L. and J. Alonso. (2018) Periostin: a matricellular protein with multiple functions in cancer development and progression. Front Oncol. 8: 225 10.3389/fonc.2018.00225 10.3389/fonc.2018.00225 PMC6005831 29946533

[142] Direnzo D et al. (2012) Induced Mist1 expression promotes remodeling of mouse pancreatic acinar cells. Gastroenterology. 143(2): 469–80 10.1053/j.gastro.2012.04.011 10.1053/j.gastro.2012.04.011 PMC3664941 22510200

[143] Pan J, Seeger-Nukpezah T, Golemis EA (2013) The role of the cilium in normal and abnormal cell cycles: emphasis on renal cystic pathologies. Cell Mol Life Sci. 70(11): 1849–74 10.1007/s00018-012-1052-z 10.1007/s00018-012-1052-z PMC3657316 22782110

[144] Mariman EC et al. (2016) The cilium: a cellular antenna with an influence on obesity risk*.* Br J Nutr 116(4): 576–92 10.1017/S0007114516002282 10.1017/S0007114516002282 27323230

[145] Davenport JR et al. (2007) Disruption of intraflagellar transport in adult mice leads to obesity and slow-onset cystic kidney disease. Curr Biol. 17(18): 1586–94 10.1016/j.cub.2007.08.034 10.1016/j.cub.2007.08.034 PMC2084209 17825558

[146] Dalbay MT et al. (2015) Adipogenic differentiation of hMSCs is mediated by recruitment of IGF-1r onto the primary cilium associated with cilia elongation. Stem Cells. 33(6): 1952–61 10.1002/stem.1975 10.1002/stem.1975 PMC4737234 25693948

[147] Marion V et al. (2009) Transient ciliogenesis involving Bardet-Biedl syndrome proteins is a fundamental characteristic of adipogenic differentiation. Proc Natl Acad Sci USA. 106(6): 1820–5 10.1073/pnas.0812518106 10.1073/pnas.0812518106 PMC2635307 19190184

[148] Forcioli-Conti N et al. (2015) The primary cilium undergoes dynamic size modifications during adipocyte differentiation of human adipose stem cells. Biochem Biophys Res Commun. 458(1): 117–22 10.1016/j.bbrc.2015.01.078 10.1016/j.bbrc.2015.01.078 25637533

[149] Kopinke D, Roberson EC, Reiter JF (2017) Ciliary hedgehog signaling restricts injury-induced adipogenesis*.* Cell. 170(2): 340–351 e12 10.1016/j.cell.2017.06.035 10.1016/j.cell.2017.06.035 PMC5617351 28709001

[150] Yamakawa D (2021). Primary cilia-dependent lipid raft/caveolin dynamics regulate adipogenesis. Cell Rep.

[151] Ashizawa N et al. (1999) The morphological changes of exocrine pancreas in chronic pancreatitis*.* Histol Histopathol. 14(2): 539–52 10.14670/HH-14.539 10.14670/HH-14.539 10212816

[152] Cano DA, Sekine S, Hebrok M (2006) Primary cilia deletion in pancreatic epithelial cells results in cyst formation and pancreatitis*.* Gastroenterology. 131(6): 1856–69 10.1053/j.gastro.2006.10.050 10.1053/j.gastro.2006.10.050 17123526

[153] Huangfu D, Anderson KV (2005) Cilia and Hedgehog responsiveness in the mouse. Proc Natl Acad Sci USA. 102(32): 11325–30 10.1073/pnas.0505328102 10.1073/pnas.0505328102 PMC1183606 16061793

[154] Cano DA et al. (2004) Orpk mouse model of polycystic kidney disease reveals essential role of primary cilia in pancreatic tissue organization. Development. 131(14): 3457–67 10.1242/dev.01189 10.1242/dev.01189 15226261

[155] Zhang Q et al. (2005) Disruption of IFT results in both exocrine and endocrine abnormalities in the pancreas of Tg737(orpk) mutant mice. Lab Invest. 85(1): 45–64 10.1038/labinvest.3700207 10.1038/labinvest.3700207 15580285

[156] Cyge B et al. (2021) Loss of the ciliary protein Chibby1 in mice leads to exocrine pancreatic degeneration and pancreatitis*.* Sci Rep. 11(1): 17220 10.1038/s41598-021-96597-w 10.1038/s41598-021-96597-w PMC8390639 34446743

[157] Kropp PA, Zhu X, Gannon M (2019) Regulation of the Pancreatic Exocrine Differentiation Program and Morphogenesis by Onecut 1/Hnf6*.* Cell Mol Gastroenterol Hepatol. 7(4): 841–856 10.1016/j.jcmgh.2019.02.004 10.1016/j.jcmgh.2019.02.004 PMC6476890 30831323

[158] Augereau C (2016). Chronic pancreatitis and lipomatosis are associated with defective function of ciliary genes in pancreatic ductal cells. Hum Mol Gene.

[159] Zhang H (2009). Multiple, temporal-specific roles for HNF6 in pancreatic endocrine and ductal differentiation. Mech Dev.

[160] Quilichini E (2019). Pancreatic ductal deletion of hnf1b disrupts exocrine homeostasis, leads to pancreatitis, and facilitates tumorigenesis. Cell Mol Gastroenterol Hepatol.

[161] Yanardag S, Pugacheva EN (2021) Primary Cilium Is Involved in Stem Cell Differentiation and Renewal through the Regulation of Multiple Signaling Pathways*.* Cells. 10(6) 10.3390/cells10061428 10.3390/cells10061428 PMC8226522 34201019

[162] Lodh S, O'Hare EA, Zaghloul NA (2014). Primary cilia in pancreatic development and disease. Birth Defects Res C Embryo Today.

[163] Golson ML et al. (2009) Ductal malformation and pancreatitis in mice caused by conditional Jag1 deletion. Gastroenterology. 136(5): 1761–71 e1 10.1053/j.gastro.2009.01.040 10.1053/j.gastro.2009.01.040 19208348

[164] Hidalgo-Sastre A (2016). Hes1 controls exocrine cell plasticity and restricts development of pancreatic ductal adenocarcinoma in a mouse model. Am J Pathol.

[165] Pearring JN (2017). Loss of Arf4 causes severe degeneration of the exocrine pancreas but not cystic kidney disease or retinal degeneration. PLoS Genet.

[166] Baumann B (2007). Constitutive IKK2 activation in acinar cells is sufficient to induce pancreatitis in vivo. J Clin Invest.

[167] Algul H (2007). Pancreas-specific RelA/p65 truncation increases susceptibility of acini to inflammation-associated cell death following cerulein pancreatitis. J Clin Invest.

[168] Li N (2013). Loss of acinar cell IKKalpha triggers spontaneous pancreatitis in mice. J Clin Invest.

[169] Nakagawa K et al. (2019) UBIAD1 Plays an Essential Role in the Survival of Pancreatic Acinar Cells. Int J Mol Sci. 20(8) 10.3390/ijms20081971 10.3390/ijms20081971 PMC6515134 31013667

[170] Bottinger EP (1997). Expression of a dominant-negative mutant TGF-beta type II receptor in transgenic mice reveals essential roles for TGF-beta in regulation of growth and differentiation in the exocrine pancreas. EMBO J.

[171] Chuvin N (2017). Acinar-to-ductal metaplasia induced by transforming growth factor beta facilitates KRAS. Cell Mol Gastroenterol Hepatol.

[172] Kong K (2020). Progress in animal models of pancreatic ductal adenocarcinoma. J Cancer.

[173] Chidawanyika T (2018). SEC24A identified as an essential mediator of thapsigargin-induced cell death in a genome-wide CRISPR/Cas9 screen. Cell Death Discov.

[174] Liu H, Kiseleva AA, Golemis EA (2018). Ciliary signalling in cancer. Nat Rev Cancer.

[175] Pugacheva EN (2007). HEF1-dependent Aurora A activation induces disassembly of the primary cilium. Cell.

[176] Tape CJ (2016). Oncogenic KRAS regulates tumor cell signaling via stromal reciprocation. Cell.

[177] Ingham PW (2022). Hedgehog signaling. Curr Top Dev Biol.

[178] Bangs FK, Miller P, O'Neill E (2020) Ciliogenesis and Hedgehog signalling are suppressed downstream of KRAS during acinar-ductal metaplasia in mouse. Dis Model Mech. 13(7) 10.1242/dmm.044289 10.1242/dmm.044289 PMC7406310 32571902

[179] Lee H (2022) Obesity-associated cancers: evidence from studies in mouse models. Cells. 11(9) 10.3390/cells11091472 10.3390/cells11091472 PMC9102145 35563777

[180] Alicea GM (2020). Changes in aged fibroblast lipid metabolism induce age-dependent melanoma cell resistance to targeted therapy via the fatty acid transporter FATP2. Cancer Discov.

[181] Fane ME (2022). Stromal changes in the aged lung induce an emergence from melanoma dormancy. Nature.

[182] Fane M, Weeraratna AT (2020). How the ageing microenvironment influences tumour progression. Nat Rev Cancer.

[183] Engle SE (2021). Cilia signaling and obesity. Semin Cell Dev Biol.

[184] Fendrich V (2008). Hedgehog signaling is required for effective regeneration of exocrine pancreas. Gastroenterology.

[185] Shi Y, Long F (2017) Hedgehog signaling via Gli2 prevents obesity induced by high-fat diet in adult mice. Elife. 6 10.7554/eLife.31649 10.7554/eLife.31649 PMC5716664 29205155

[186] Morris JPt, Wang SC, Hebrok M, (2010). KRAS, Hedgehog, Wnt and the twisted developmental biology of pancreatic ductal adenocarcinoma. Nat Rev Cancer.

[187] Steele NG (2021). Inhibition of hedgehog signaling alters fibroblast composition in pancreatic cancer. Clin Cancer Res.

[188] Hosein AN, Brekken RA, Maitra A (2020). Pancreatic cancer stroma: an update on therapeutic targeting strategies. Nat Rev Gastroenterol Hepatol.

[189] Halbrook CJ, Lyssiotis CA (2017) Employing Metabolism to Improve the Diagnosis and Treatment of Pancreatic Cancer. Cancer Cell. 31(1): 5–19 10.1016/j.ccell.2016.12.006 10.1016/j.ccell.2016.12.006 28073003

[190] Golemis EA et al (2018) Molecular mechanisms of the preventable causes of cancer in the United States. Genes Dev. 32(13–14): 868–902 10.1101/gad.314849.118 10.1101/gad.314849.118 PMC6075032 29945886

[191] Jennings RE (2013). Development of the human pancreas from foregut to endocrine commitment. Diabetes.

